# A synthetic gut microbiota provides an understanding of the maintenance and functional impact of phage

**DOI:** 10.1128/mbio.02341-25

**Published:** 2025-11-12

**Authors:** Heejung Koo, Kerim Heber, Shuchang Tian, Shane T. Connolly, Fuhua Hao, Jingcheng Zhao, Bethany Swencki-Underwood, Andrew D. Patterson, Guy E. Townsend, Jordan E. Bisanz

**Affiliations:** 1 Department of Biochemistry and Molecular Biology, Pennsylvania State University 189499, University Park, Pennsylvania, USA; 2 Department of Veterinary and Biomedical Sciences, Center for Molecular Toxicology and Carcinogenesis, The Pennsylvania State University 311374, University Park, Pennsylvania, USA; 3 One Health Microbiome Center, Huck Life Sciences Institute, University Park, Pennsylvania, USA; 4 Department of Molecular and Precision Medicine, Penn State College of Medicine 12310 , Hershey, Pennsylvania, USA; University of Pittsburgh School of Medicine, Pittsburgh, Pennsylvania, USA

**Keywords:** gut microbiome, bacteriophages, synthetic communities, metagenomics, gnotobiotics

## Abstract

Phages are under intense study as therapeutics and mediators of microbial community behavior; however, tractable models are needed to study phages in the context of the mammalian gut. To address this gap, we isolated phages against members of a synthetic gut microbial community (sFMT), identifying the *Bacteroides uniformis* JEB00023 (DSM 6597) phage HKP09. While resistance to HKP09 was observable within hours of infection in monoculture, high titers of HKP09 were maintained *in vitro* and in gnotobiotic mouse models over extended periods. Sequencing of resistant *B. uniformis* lines revealed phase variation upstream of a capsular polysaccharide locus driving the generation of resistant and sensitive subpopulations, thus demonstrating a mechanism allowing stable coexistence of both virus and bacterial host. Communities infected *in vitro* and *in vivo* with HKP09 showed transiently reduced loads of *B. uniformis* DSM 6597. Its impact in the gut was distinct from communities constructed without its host *B. uniformis* strain (sFMT∆JEB00023). Rather than a compensatory increase in closely related *Bacteroides* strains, the most significant impacts were observed on distantly related strains, demonstrating that phage perturbations more broadly impact community structure in ways not easily predicted by phylogeny or simple strain exclusion. Metabolomic analyses of the feces of HKP09-infected sFMT-colonized gnotobiotic animals demonstrated altered abundances of amino acids and microbial fermentation products compared to uninfected mice and those colonized with sFMT∆JEB00023. Taken together, these data provide a controlled model for studying phages in the context of the mammalian gut, providing mechanistic insights into phage-host dynamics and their consequences on the function of microbial communities.

**IMPORTANCE:**

Phages are key members of the gut microbiome, but the understanding of their biological significance for host health lags behind their bacterial hosts. In this study, we demonstrate the use of a phage-infection model using defined, synthetic microbial communities that colonize the intestinal tract of mice. We uncovered that spontaneous inversions in the genome of *Bacteroides uniformis* perpetually generate subpopulations, which are either sensitive or resistant to phage infection, allowing for the coexistence of predator and prey in this species. Phage infection demonstrated broad impacts on community structure and metabolism in animals, which are not easily predicted by the exclusion of the viral host. This research demonstrates a tractable approach through which the impacts of phage on both the microbiome and mammalian host can be deciphered.

## INTRODUCTION

The human virome is the collection of viruses present in and on the body; however, the majority of nucleic acid constituting the virome is not mappable to reference databases and referred to as “viral dark matter.” This unknown nature of the virome underscores a need for characterization and elucidation of its biological functions. This will be essential to understand how phages of the virome modulate the composition and function of microbial communities to shape human health. While shifting phage populations have been associated with inflammatory bowel disease, diabetes, colorectal cancer, and other diseases (1–8), experimental models to establish causality and mechanisms are currently limited due to the specific host range of phages, rapid rise of resistance, and limited commensal-targeting phages available in public repositories.

Phage and their host coevolve and coexist through a variety of means and can be classed based on the outcome of an infection. Lytic phages infect their host, replicate, and lyse the cell to break free. Lysogenic phages integrate their genome into the host and repress their induction, replication, and lysis until a time of stress. Finally, some phages replicate within a host without lysing it, instead of producing budding progeny. These mechanisms dictate the selective pressure on the host bacteria to adapt their genomes, as well as the ability for the phage to coexist with its host. Typically, bacteria will respond to lytic phage through mutations in an evolutionary arms race (9, 10); however, recent research has shown that phase variation (preprogrammed and reversible genetic alterations) plays a major role in phage-host dynamics for *Bacteroides* species in the gut (11–13). Through stochastic inversions, a bacterial strain may maintain a sensitive subpopulation of the host that feeds a persistent population of phage. This mechanism has been described in *Bacteroides thetaiotaomicron, B. intestinalis*, and *B. xylanisolvens* species, mostly with Caudoviricetes and *crAss*phages (11–15). This is ecologically significant as a mechanism allowing for a long-term selective pressure that may lead to maintained functional microbiome alterations.

We sought to create a tractable model to investigate the overall compositional and functional effects of persistent phage infection. We isolated a strain-specific Caudoviricetes phage infecting the type strain of *B. uniformis*, one of the most abundant and prevalent species of the human gut microbiome. We found the bacterial host maintains a persistent phage population by controlling resistance through phase variation, which we observed in *B. uniformis* metagenomes-assembled genomes (MAGs) from across the globe. We used a 38-strain synthetic human gut microbial community we previously characterized as a host community, uncovering that phage infection and persistence shaped global microbiota composition and metabolism in ways that could not be explained due to the simple exclusion of the host strain. Models such as these will be required to perform mechanistic experiments to understand how phages shape the function of microbial communities to influence host health.

## RESULTS

### Isolation and genomic characterization of bacteriophage HKP09

**Fig 1:**
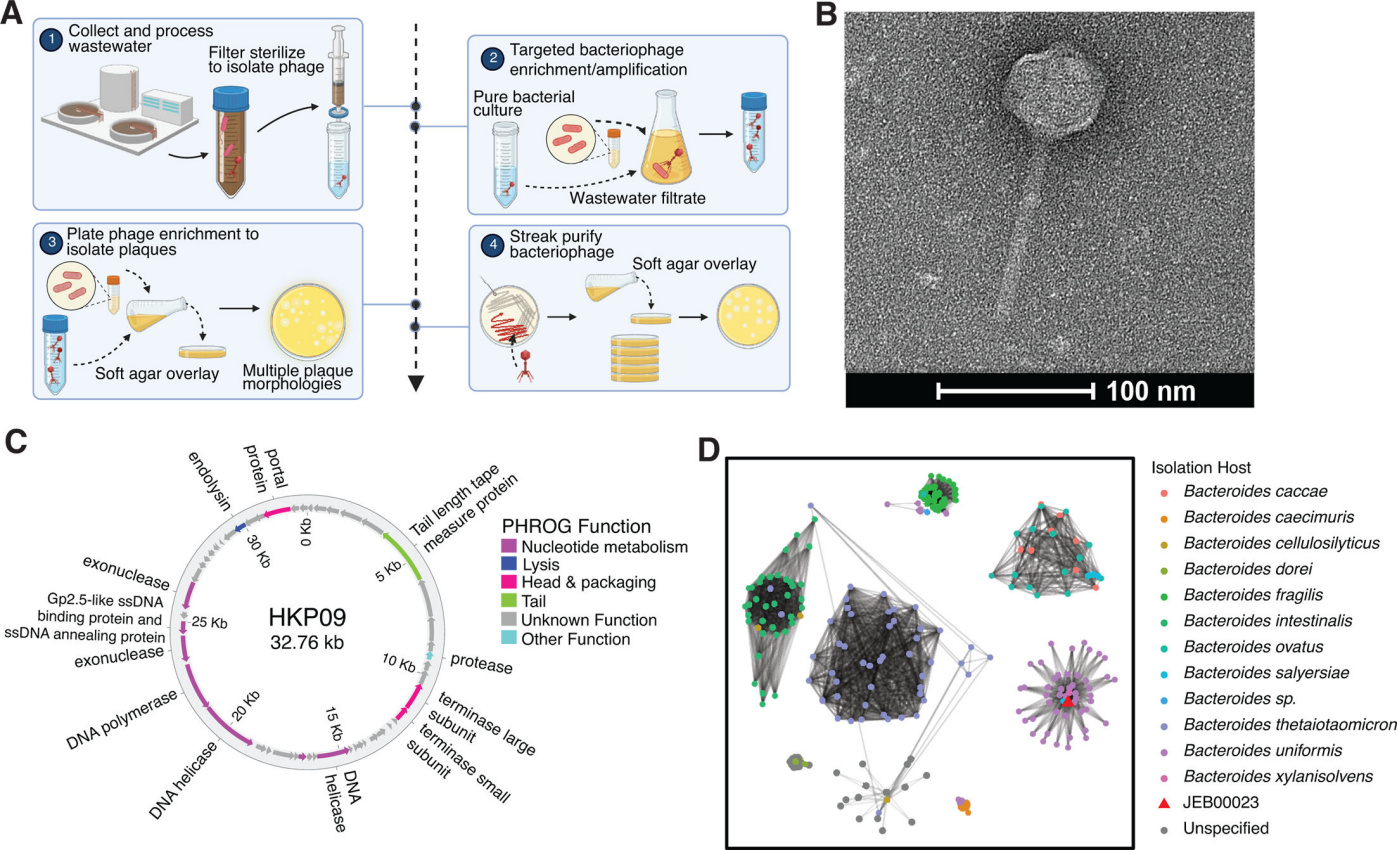
Isolation and characterization of the representative *B. uniformis* phage HKP09. (**A**) HKP09 was isolated from wastewater using an enrichment approach. (**B**) Transmission electron microscopy of HKP09 demonstrates a tailed phage with a tail length of 114 ± 9 nm, a head length of 63 ± 3 nm, and a head width of 62 ± 1 nm (*N* = 6 phage particles, mean ± sd). (**C**) HKP09 has a 32.76 kb genome with hallmark viral genes. (**D**) Genomic comparison of HKP09 against previously isolated phages demonstrates it is representative of those previously isolated against *B. uniformis* (*N* = 170 *Bacteroides* phages analyzed). Nodes represent phages, which are colored by isolation host. Edges represent significant gene-content similarity between viral genomes, as determined by vConTACT2 using default parameters.

We first sought to isolate lytic phages targeting members of sFMT through enrichment cultures and plaquing assays. We leveraged wastewater collected from a local sewage plant as a rich source of human gut phages (Fig. 1A). After purification, we obtained the phage HKP09 from cultures of our line of *Bacteroides uniformis* DSM 6597 (termed JEB00023). Transmission electron microscopy demonstrated that HKP09 is a tailed phage with a tail length of approximately 114 nm and a symmetrical capsid of approximately 62 nm in both dimensions (Fig. 1B). Long-read sequencing and analysis of the HKP09 genome demonstrated that it has a double-stranded DNA genome of approximately 32.76 kb (Fig. 1C). The HKP09 genome contains 50 open reading frames, of which 37 are of unknown function, and the remainder belong to structural components and hallmark genes for phage replication. To contextualize the similarity of HKP09 to other *Bacteroides* phages and more confidently assign taxonomy, we conducted a gene sharing similarity analysis (16) against a collection of phages previously described to have been isolated on various *Bacteroides* species (17). This analysis confirmed that HKP09 is a cultured representative of a larger clade within the Caudoviricetes class that has been previously isolated from *B. uniformis* strains (Fig. 1D).

Comparison of HKP09 to high-quality viral genomes from the Unified Human Gut Virome (UHGV) Catalog (18) demonstrated that HKP09-like phages are present in 9% of individuals (249 of 2,758) of samples with detection in 16 countries representing all contents. Detection was highest in China (15.8% samples) with a detection in 6.5% of samples from the United States. Detection was not significantly associated with gender, age, or body mass index (*P* > 0.05 Fisher’s exact test and Mann-Whitney U test). Taken together, these results demonstrate that HKP09 is a representative of a common phage found in the human gut, warranting further study in tractable experimental systems.

### Phenotypic resistance to HKP09 is associated with phase variation

**Fig 2:**
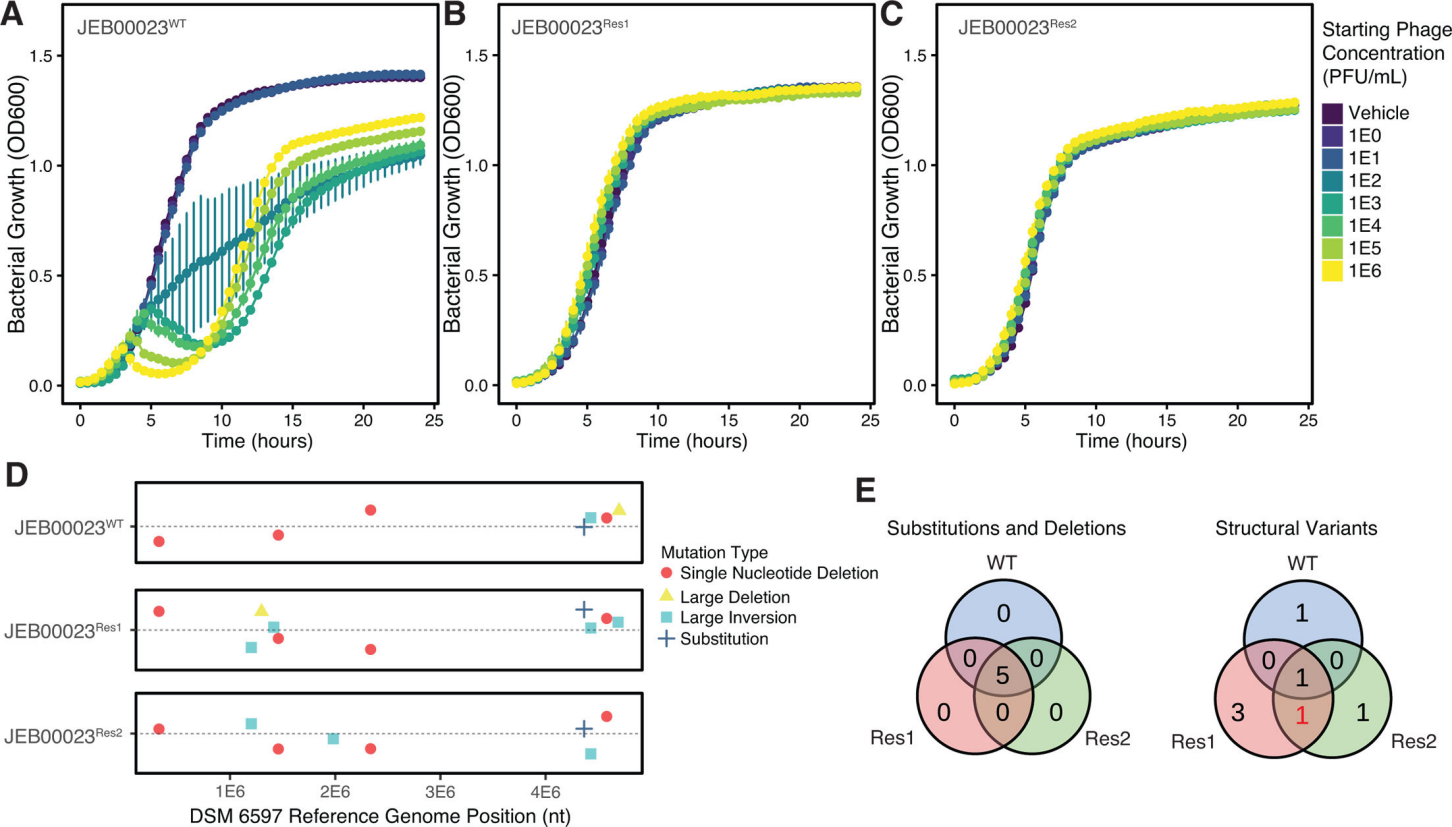
Structural variants are associated with spontaneous phage resistance. (**A**) Growth curves of the wild-type *B. uniformis* DSM 6597 (JEB00023^WT^) co-cultured with HKP09 show a concentration-dependent inhibition of growth with the emergence of resistant populations within 10 hours of infection. (**B, C**) Resistant isolates JEB00023^Res1^ and JEB00023^Res1^ were isolated from resistant populations depicted in panel A and challenged again with a gradient of HKP09, demonstrating no growth defects. Error bars in panels A–C represent mean ± standard error of three replicates at each concentration of phage. (**D**) Hybrid sequencing of the parental and spontaneously resistant isolates demonstrates a limited number of mutations compared to the reference DSM 6597 sequence. Points are jittered vertically to allow visualization. (**E**) Venn diagrams show shared mutations and structural variants. Only one mutation was observed to be exclusively shared by JEB00023^Res1^ and JEB00023^Res2^: a large inversion located centered at 1,293,126 nt upstream of a putative capsular polysaccharide synthesis locus.

Infection assays with HKP09 and *B. uniformis* DSM 6597 (JEB00023^WT^) demonstrated rapid emergence of HKP09-resistant populations across a wide range of viral inocula up to the maximum assayed multiplicity of infection of approximately 1 (Fig. 2A). To determine whether resistance was sustained, isolated colonies from these resistant populations were challenged with HKP09, and no impact on microbial growth was noted (Fig. 2B and C). To understand potential mutations responsible for HKP09 resistance, we re-sequenced our DSM 6597 isolate (JEB00023^WT^) and two resistant isolates (JEB00023^Res1^ and JEB00023^Res2^). Through a hybrid sequencing approach combining high-coverage Illumina short reads and Nanopore long reads, we detected genetic variants associated with HKP09 resistance (Fig. 2D). Our parental line (JEB00023^WT^) was found to contain seven variants relative to the reference genome. While no single-nucleotide substitutions or insertion/deletion mutations were associated with HKP09 resistance, a single shared structural variant was observed in the resistant populations (Fig. 2E).

**Fig 3:**
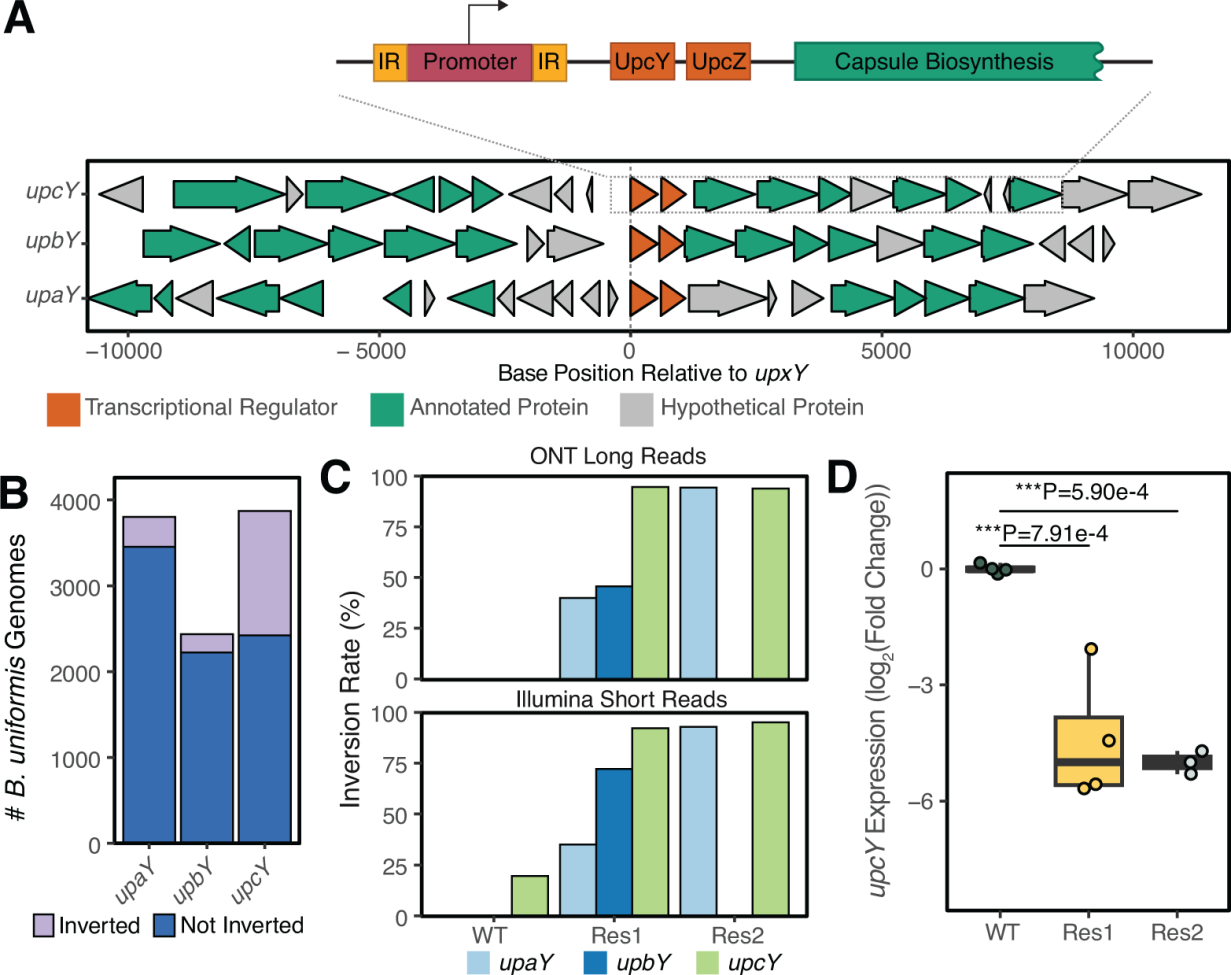
*B. uniformis* populations display phase variation upstream of the *upcY* locus, impacting the expression of downstream genes. (**A**) The inversion associated with resistant *B. uniformis* strains lies upstream of the *upcY-associated* capsular polysaccharide locus, one of three phase-variable *upx*Y-family-associated regions in the DSM 6597 genome. (**B**) Inversion rates at each *upxY-*associated locus in MAGs assigned to *B. uniformis* demonstrate that resistant populations exist in natural host-associated populations (UHGG v2.0 database, inversion relative to reference DSM 6597 genome). (**C**) Genome sequencing using both long read (46.67×– 126.12× coverage) and short read (82.93×–91.50× coverage) of isolates demonstrates that phase variation drives genetic heterogeneity within pure cultures. (**D**) qPCR analysis demonstrates that inversion upstream of *upcY* in JEB00023^Res1^ and JEB00023^Res2^ significantly decreases the expression of *upcY* transcripts. *N* = 4 replicate cultures/condition, expression normalized to *gapdh*. Statistical analysis by ANOVA with Tukey HSD.

A closer analysis of the shared variant revealed a 174 nucleotide inversion flanked by 27 nucleotide inverted repeat (IR) sequences (CGTCCATTAAACGAACGTTTAAAAAAC; Fig. 3A). This locus, which we identified as the *upcY* locus, contains an operon of genes involved in capsular polysaccharide biosynthesis. Preceding the polysaccharide synthesis genes is UpcY, an antitermination factor, and UpcZ, an inhibitor of UpxY at other loci. These genes are known to regulate capsular polysaccharide biosynthesis in other *Bacteroides* spp. (19). On the basis of homology searches, we identified three such loci in the *B. uniformis* DSM 6597 reference genome. Phase variation at these loci driven by inversions has been previously described to determine phage tropism in other *Bacteroides* spp. through a mechanism involving differential expression of capsular polysaccharide genes leading to altered capsule composition, which impacts phage infectivity (11, 15). To understand the frequency at which phase variation at these loci exists in natural host-associated *B. uniformis* populations, we analyzed *B. uniformis* genomes present in the UHGG database, which is primarily composed of MAGs (20). Of the 6,001 *B. uniformis* genomes queried, inversions relative to JEB00023^WT^ were noted at 9.16%, 8.74%, and 37.4% of *upaY, upbY,* and *upcY* loci identified (Fig. 3B). Given potential issues with contamination in MAGs, we replicated the analysis in 262 isolate genomes and observed similar values of 11.7%, 14.0%, and 29.6% inversion at *upaY, upbY,* and *upcY* loci, providing an approximation of the greater sampling. Based on these observations, we hypothesized that immediate resistance to HKP09 was dictated by pre-existing inverted subpopulations rather than new mutations. Using both short- and long-read approaches, we identified variable and mixed inversions at all three UpxY-associated loci within our parental and resistant isolates (Fig. 3C). Notably, in our parental isolate, 19.5% of the population already exhibited inversion at the *upcY* locus, providing a plausible explanation for the rapid emergence of resistance to HKP09. To confirm that the inversion did impact expression of the downstream *upcY* anti-terminator, we leveraged reverse transcription (RT)-qPCR, observing significantly reduced expression during the mid-exponential growth in both resistant isolates (Fig. 3D).

### Deletion of IRs modifies susceptibility to infection

**Fig 4:**
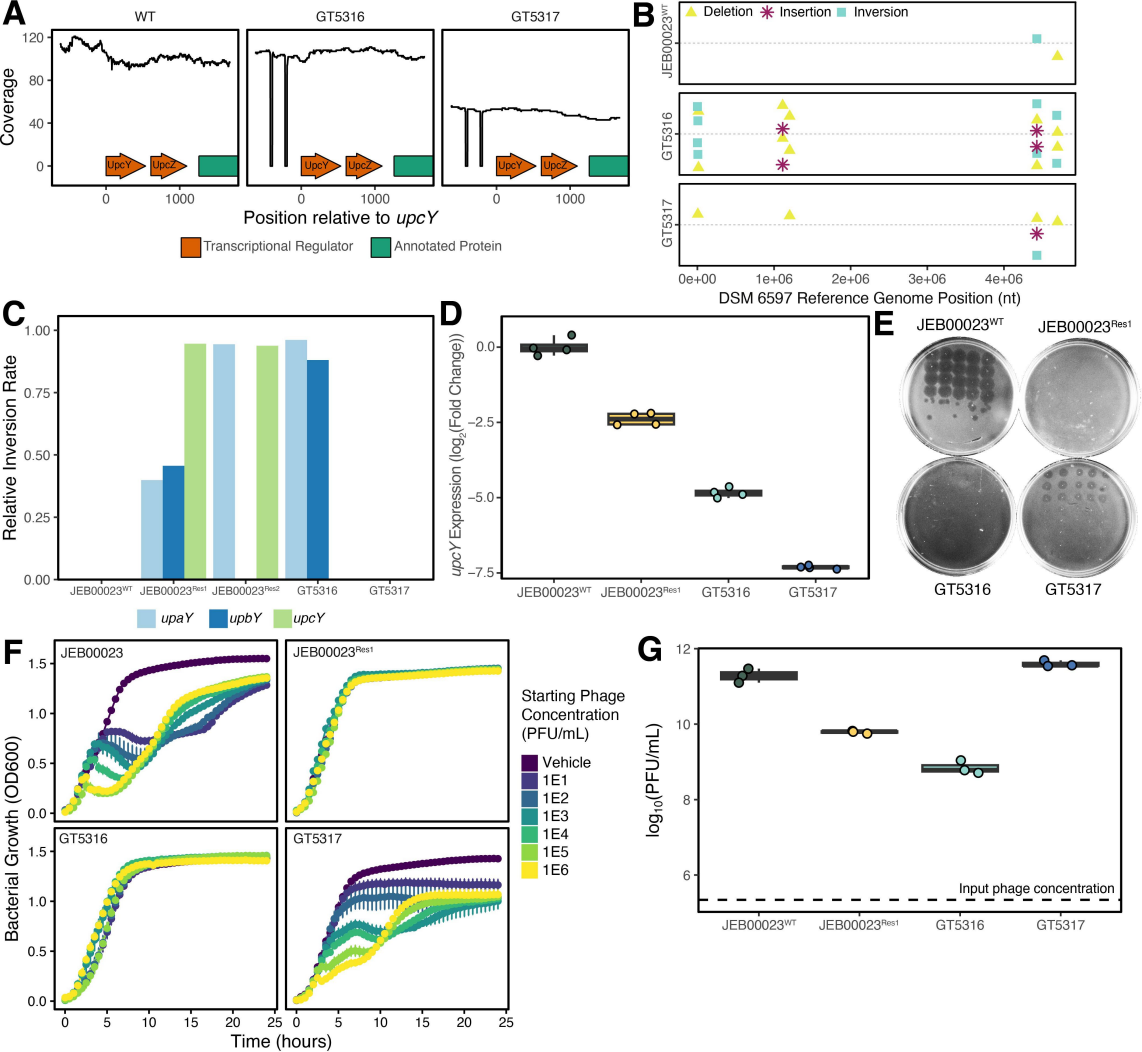
IR deletion mutants display variable HKP09 resistance. (**A**) Long-read sequencing of the UpcY region demonstrates two mutants with deleted IRs. (**B**) The two mutants have few mutations apart from the deleted IRs. (**C**) Long-read sequencing demonstrates that GT5317 shares the same phase inversion rates as JEB00023^WT^, and GT5316 has the other two loci primarily inverted. (**D**) qPCR of UpcY RNA shows decreased, yet distinct, expression in the deletion mutants. (**E**) Through spot titer assays, JEB00023^WT^ and GT5317 express the sensitive phenotype, whereas GT5316 and JEB00023^Res1^ are resistant. (**F**) Growth curves further demonstrate that HKP09 infects JEB00023^WT^ and GT5317, and alters growth kinetics. Both strains show the ability to develop resistance within 15 hours. GT5316 shows resistance to HKP09 infection. Error bars represent mean ± standard error. (**G**) Phage titers after 24 hours of phage and strain co-cultures (1E5 PFU/mL starting concentration) demonstrate that all strains allowed for phage replication, with resistant strains maintaining a smaller population.

To better understand how phase variation mediates predator-prey dynamics between HKP09 and *B. uniformis* DSM 6597, we generated deletions of both IRs at the *upcY* locus, which were confirmed through genome re-sequencing (Fig. 4A). We hypothesized that this would disable rearrangement at the *upcY* locus and interfere with UpcY’s anti-termination activity by disrupting its promoter. Two knockout isolates were successfully obtained: GT5316 and GT5317, which differed in inversions affecting the other *upx* loci (Fig. 4B and C). Notably, GT5317 exactly mirrors the wild-type arrangement at the other *upx* loci, while GT5316 shows rearrangement at the loci for *upcY* and *upbY*. qPCR analysis of the *upcY* anti-terminator downstream of the deletions revealed significantly reduced expression below levels found in the spontaneously resistant JEB00023^Res1^ population (Fig. 4D). Through both plaquing and broth infection assays, we found that the GT5316 exhibited pre-existing resistance to HKP09, while the GT5317 isolate demonstrated an intermediate phenotype (Fig. 4E and F). Plaque morphology on GT5317 was distinct from that of the wild-type strain, exhibiting a less defined zone of clearing, which is a phenomenon previously observed in *B. thetaiotaomicron* phage when their host has a variable set of capsular polysaccharides (11). Finally, through quantifying the resulting phage titer after infection in liquid culture, we observed that while all lines of *B. uniformis* DSM 6597 could propagate HKP09, significantly lower titers were recovered from the spontaneously resistant JEB00023^Res1^ and GT5316 isolates. Taken together, these results demonstrate that phase variation mediates susceptibility to HKP09. However, resistance is not a binary phenotype, nor is it absolute, given that all mutant strains could propagate HKP09, even in the absence of altered growth kinetics (Fig. 4G). These data further demonstrate that the resistance mechanism is epistatic, likely resulting from interactions across *upx* and/or other loci. Additional work is required to map phage receptors to understand whether phase variation is altering receptor expression or shielding receptors through varied capsular polysaccharides.

### HKP09 shapes bacterial inter- but not intra-species competition *in vitro*

**Fig 5:**
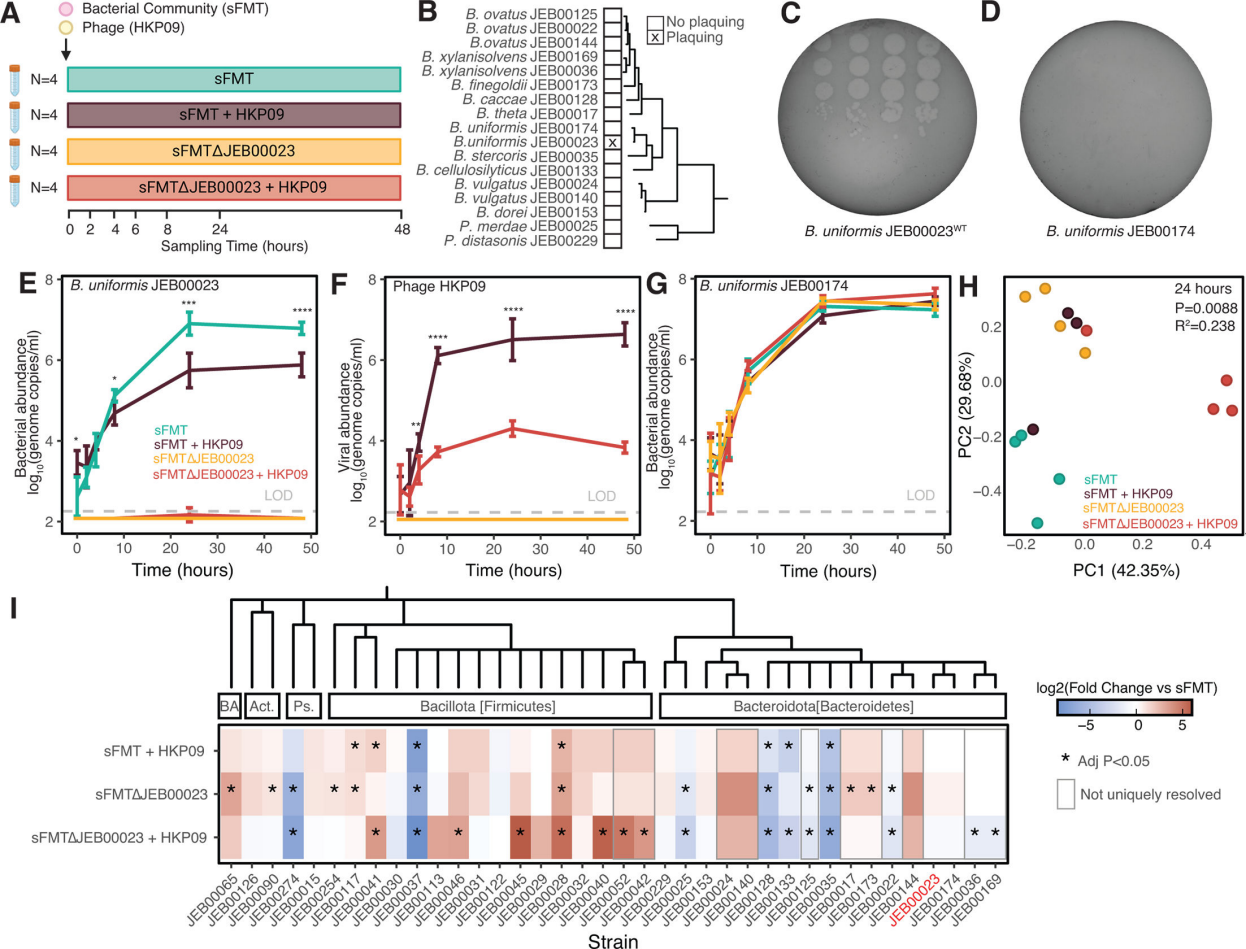
HKP09 is maintained in a complex community *in vitro*. (**A**) Experimental design of *in vitro* community infection experiment (*N* = 4 culture replicates/condition). (**B**) Plaquing assays against all Bacteroidaceae in sFMT demonstrate that HKP09 only plaques on *B. uniformis* JEB00023 (DSM 6597). Strains are organized by whole-genome phylogeny. Representative plaquing assays for (**C**) HKP09-sensitive *B. uniformis* JEB00023^WT^ and (**D**) resistant *B. uniformis* JEB00174. (**E**) Abundance of *B. uniformis* JEB00023 is decreased by an order of magnitude in response to infection with HKP09. (**F**) Phage HKP09 productively replicates during infection. (**G**) *B. uniformis* JEB00174 is not meaningfully impacted by the presence of HKP09 or the exclusion of JEB00023 from the synthetic community. *N* = 4 replicates/group in panels E–G. Statistical analysis by ANOVA with Tukey HSD with asterisks denoting significance between sFMT and sFMT + HKP09 groups. (**H**) Principal component analysis (PCA) after 24 hours indicates distinct compositional differences between experimental groups (PERMANOVA *P* = 0.0088, R^2^ = 0.2382). (**I**) 24 hours after infection, phylogenetically diverse strains are impacted by the presence of HKP09, which mimics the effect of exclusion of JEB00023. BA Bacillota_A, Act. Actinomycetota (Actinobacteria), Ps. Pseudomonadota (Proteobacteria). Strains that cannot be resolved from each other are shown in gray boxes. Statistical analysis by ANOVA with Dunnett’s Test compared to sFMT control. * denotes an adjusted *P*-value < 0.05. The tree represents a cladogram based on taxonomy.

We next sought to determine how the inclusion of HKP09 shapes community dynamics using an *in vitro* culture system (Fig. 5A). We utilized the full 38-member synthetic community sFMT (Table S1), and a variant constructed without the HKP09-host strain JEB00023/DSM 6597 (sFMT∆JEB00023) as a control. Among all members of sFMT, HKP09 appears to be specific for *B. uniformis* JEB00023 as determined through plaquing assays (Fig. 5B; Fig. S1A), including the closely related *B. uniformis* JEB000174 (Fig. 5C and D). Given that the GT5316 mutant was capable of propagating HKP09 in the absence of plaquing, we also confirmed that *B. uniformis* JEB00174 could not propagate HKP09 through qPCR, finding no evidence of phage replication (Fig. S1B). Using strain-specific qPCR assays, we noted that the inclusion of HKP09 in the culture system resulted in productive infection, leading to a log reduction in the absolute abundance of JEB00023 after 24 hours of co-culture (Fig. 5E). This was accompanied by sustained high titers of HKP09 in solution at a phage-to-host ratio of 7.3:1, as measured by qPCR-derived genome copies (Fig. 5F). While we initially hypothesized that reductions in JEB00023 would be compensated by its nearest relative *B. uniformis* JEB00174, due to presumed similar niches, no significant differences were observed in JEB00174 abundance (Fig. 5G). We next leveraged V4 16S rRNA amplicon sequencing to examine community composition (Fig. 5H). We found that the inclusion of HKP09 and the absence of JEB00023 had significant effects on community composition (*P* = 0.0088, R^2^ = 0.238, PERMANOVA). Interestingly, we found that no single clade compensates for the reduction/absence of JEB00023, and that no other *Bacteroides* spp. significantly increased in abundance after HKP09 infection. Alternatively, some of the most impacted strains were the Bacillota (Firmicutes) *Anaerobutyricum hallii* JEB00037, *Enterocloster asparagiformis* JEB00028, and *Faecalibacterium prausnitzii* JEB00041 (Fig. 5I; Fig. S2). These changes were largely mirrored by the intentional exclusion of the host JEB00023 (sFMT∆JEB00023), albeit with more strains being significantly impacted by its complete absence. Complete analysis of per-strain abundances is found in Fig. S2.

### HKP09 infection shapes community composition and metabolism *in vivo*

**Fig 6:**
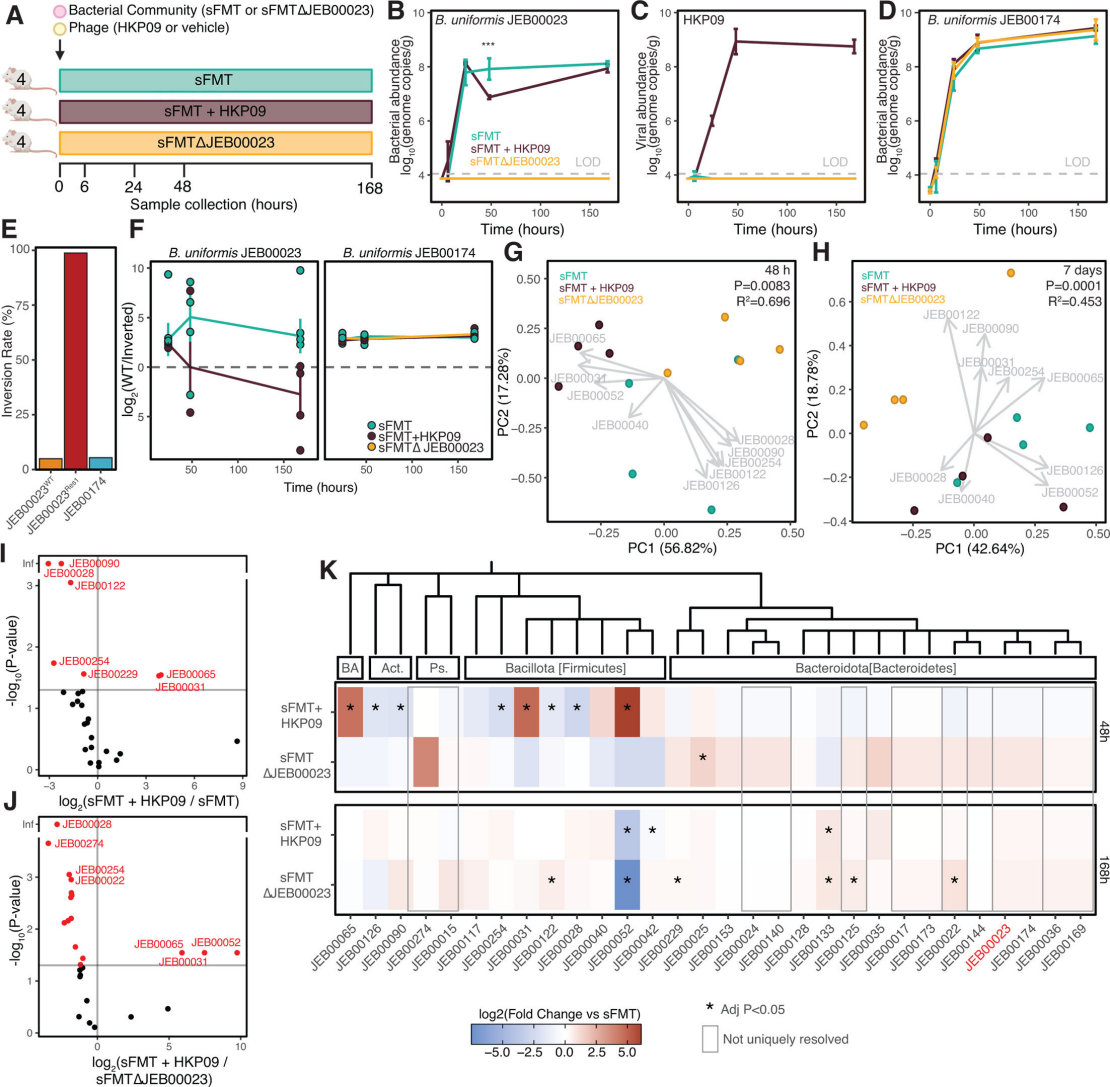
HKP09 infection is sustained *in vivo,* impacting global microbiota composition. (**A**) Experimental design for infection of sFMT in gnotobiotic mice (*N* = 4 mice/group). (**B**) *B. uniformis* JEB00023 abundance is transiently decreased in the presence of HKP09 at 48 hours compared to the vehicle control. Statistical analysis between sFMT and sFMT + HKP09 by ANOVA with Tukey HSD. (**C**) HKP09 reaches peak viral load at 48 hours and is maintained throughout 7 days. (**D**) *B. uniformis* JEB00174 is not significantly impacted by HKP09 infection. (**E**) Inversion rates at the *upcY* locus determined from amplicon sequencing concur with data from long- and short-read sequencing whole-genome sequencing. (**F**) While inversion rates at the *B. uniformis* JEB00174 *upcY* locus are constant over time *in vivo*, JEB00023 shows selection for inversion. Principal component analysis (PCA) of 16S rRNA amplicon data demonstrates that resulting communities are distinct at both (**G**) 48 hours and (**H**) 7 days post-infection (*P* = 0.0083, R^2^ = 0.453; *P* = 0.0001 R^2^=0.696; PERMANOVA). Gray arrows indicate the loadings for strains that contribute to separation. (**I**) A volcano plot of strains with differential abundance as a function of HKP09 infection at 48 hours (red strains FDR < 0.1, ALDEx2). (**J**) 15 strains are differentially abundant between HKP09-infected sFMT1 as compared to sFMT1 constructed to exclude *B. uniformis* JEB00023 (sFMT∆JEB00023), indicating that the impact of phage infection is distinct from the simple exclusion of the viral host strain. (**K**) 48 and 168 hours after infection, phylogenetically diverse strains are impacted by the presence of HKP09, which is distinct from the exclusion of JEB00023. BA Bacillota_A, Act. Actinomycetota (Actinobacteria), Ps. Pseudomonadota (Proteobacteria). Strains which cannot be resolved from each other are shown in gray boxes. Statistical analysis by ANOVA with Dunnett’s Test compared to the control. * denotes an adjusted *P*-value < 0.05.

While *in vitro* systems are valuable tools, our previous work with sFMT has demonstrated that community dynamics in culture are distinct from those that take place in the mammalian gut (21). We colonized gnotobiotic mice with sFMT, sFMT + HKP09, or sFMT∆JEB00023 for up to 7 days (168 hours), sampling community composition from feces (Fig. 6A). Infection with HKP09 did not impact the abundance of *B. uniformis* JEB00023 in the first 24 hours of colonization; however, a significant reduction in abundance was noted at 48 hours demonstrating a transient population reduction (Fig. 6B). Despite this, by 7 days post-colonization, the abundance of JEB00023 was not significantly different from the uninfected sFMT control. The abundance of HKP09 continued to rise during the first 2 days of colonization, with the population maintained throughout the week of colonization with an average ratio of 8.6:1 with JEB00023, an approximation of the viral burden observed *in vitro* (Fig. 6C). There was no detectable difference in the abundance of *B. uniformis* JEB00174 as a function of HKP09 infection, or the exclusion of *B. uniformis* JEB00023 (Fig. 6D).

To understand whether phase variation may drive the persistence of HKP09 *in vivo*, we developed a custom amplicon assay targeting the *upcY* inversion, which could both differentiate the loci of *B. uniformis* JEB00023 and JEB00174 and determine their orientations (see Materials and Methods). Replicating our whole-genome sequencing data, we observed inversions in 4.918% and 5.385% of *B. uniformis* JEB00023^WT^ and JEB00023^Res^, respectively (Fig. 6E). JEB00174 exhibits a similar frequency of inversion as JEB00023^WT^; however, it does not appear to be a natural host for HKP09. Selection for inversion at the JEB00023^WT^ *upcY* locus was observed starting at 48 hours after infection, mirroring the crash and recovery of JEB00023, while the rate of inversion was stable in JEB00174 (Fig. 6F).

Analysis of community composition by 16S rRNA gene sequencing 48 hours post-colonization (Fig. 6G) and 7 days post-colonization (Fig. 6H) revealed that HKP09 shaped community composition in a distinct fashion from the sFMT∆JEB00023 community. By 7 days post-infection, the sFMT and sFMT + HKP09 groups began to converge, likely due to the rise of resistant JEB00023 populations. As observed *in vitro*, the impact of HKP09 infection was most notable in more distantly related strains, with no significant differences observed among *Bacteroides* spp. (Fig. 6I through K). Among the most HKP09-enriched strains 48 hours post-infection were *Clostridium spiroforme* JEB00065, *Clostridium scindens* JEB00031, and *Blautia producta* JEB00052, which were also the most differentially abundant strains contrasting sFMT + HKP09 and sFMT∆JEB00023 (Fig. 6J). These shifts were largely reverted by 7 days post-infection; however, the *B. producta* strain decreased in abundance, and there was a subsequent enrichment in *B. cellulosilyticus* JEB00133 (Fig. 6K). Complete analysis of per-strain abundances is found in Fig. S3. Because 16S rRNA sequencing cannot uniquely resolve all strains in the community, including many of the *Bacteroides* strains, we replicated the experiment contrasting sFMT and sFMT + HKP09 groups again with newly prepared aliquots of sFMT and HKP09, this time using metagenomic sequencing combined with strain-level tracking (22). We sequenced 1, 2, and 7 days post-colonization (Fig. S4). We could quantitatively analyze 26 of the strains in the mice, although all strains were detected in the input, and reads from fecal samples could be mapped to all strains except *C. spiroforme* JEB00065, suggesting it may not have been viable in the newly prepared sFMT aliquots. Nonetheless, we replicated our previous findings, including noting only a transient decrease in *B. uniformis* JEB00023 abundances. Notably, no significant differences were observed in the abundances of other *Bacteroides* or *Parabacteroides* species with the exception of *B. thetaiotaomicron*; however, the effect was modest (estimate = 0.075 ± 0.032 log_2_[fold-change]/day). Despite variable strain engraftment between experiments, we again observed significant effects of HKP09 on the abundance of *C. scindens, E. lenta,* and *P. russellii,* three distantly related and functionally significant strains involved in the metabolism of amino acids, bile acids, and xenobiotics.

**Fig 7:**
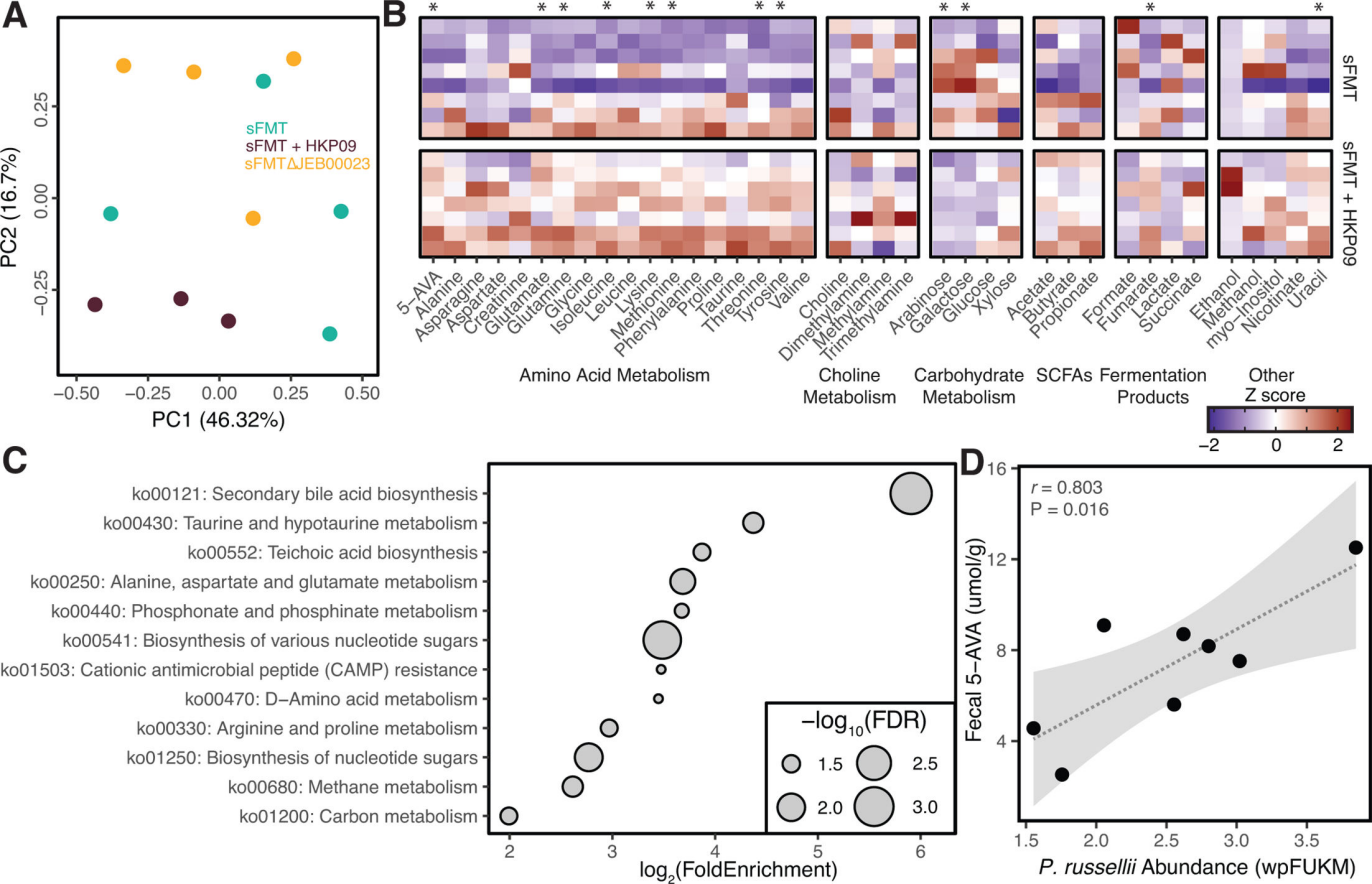
HKP09 infection results in a fecal metabolomic signature distinct from the absence of the viral host strain. (**A**) PCA of an NMR panel of 39 metabolites demonstrates that HKP09-infected communities cluster separately from the uninfected sFMT and sFMT∆JEB00023 communities (*N* = 3-4 mice/group; 48-hour time point). (**B**) Analysis of metabolite abundances demonstrates widespread impacts of HKP09 infection on microbiota metabolism, including significant effects on amino acid metabolism (*N* = 7-8 mice/group, pooled from two independent experiments, statistical analysis by Welch’s t-test). (**C**) KEGG pathway enrichment analysis of differentially abundant gene families provides evidence of correspondence between shifting microbiota community structure and metabolic outputs (FDR < 0.1). (**D**) Indirect effects of HKP09 on *P. russellii* abundance likely mediate altered 5-AVA production from proline (*P* = 0.016, Pearson correlation; wpFUKM weighted percentile fragments per thousand unique k-mers per million reads mapped).

To understand the functional consequences of HKP09 on sFMT, we used an ^1^H NMR metabolomics protocol on 48 hours post-colonization samples, uncovering distinct metabolite profiles (Fig. 7A). Mice colonized with sFMT vs sFMT + HKP09 clearly separated on the second axis of the principal component analysis (PCA); however, the sFMT and sFMT∆JEB00023 groups could not be differentiated. To identify metabolites whose abundances were impacted by HKP09 infection, we analyzed data from both experiments, revealing 12 differentially abundant metabolites (Fig. 7B). Changes in amino acid metabolism were particularly evident, including increased abundances of glutamate, glutamine, isoleucine, lysine, methionine, threonine, and tyrosine. The proline fermentation product 5-aminovalerate (5-AVA) was also increased. Additional impacts on carbohydrate and nucleotide metabolism, and the fermentation product fumarate, were also observed. To understand the extent to which shifting strain abundances could explain the metabolite pool, we conducted a differential abundance and enrichment analysis of KEGG orthologs found across the sFMT genomes (Fig. 7C; Table S2). We observed a high degree of concordance between differentially abundant metabolites and gene families which were largely driven by *B. uniformis*, *C. scindens,* and *P. russellii* strains (Table S2). These included predicting altered metabolism of proline (5-AVA), glutamate, arabinose, and threonine (Fig. 7B and C; Table S2). Our previous work has demonstrated that *P. russellii* is the primary producer of 5-AVA in sFMT when colonized in the mouse gut ([21]). Indeed, we observed that HKP09 infection results in an increased abundance of *P. russellii,* which was correlated with 5-AVA abundance, providing an intriguing example of how phage may indirectly shape metabolic outputs of distantly related strains through reshaping community dynamics (Fig. 7D).

## DISCUSSION

The use of a defined synthetic community offers a reductionist approach to dissecting the role of phages in mediating microbiota function. By controlling composition, both bacterial and viral, combined with known genomic sequences for each member of the community, we provide a bridge between what is known to occur in monoxenic models and what has been shown to occur in undefined microbial communities. We show that a challenge with a single phage results in global microbial population changes impacting phylogenetically distant strains. Surprisingly, the decrease in abundance of the host *B. uniformis* JEB00023 (DSM 6597) was not reciprocated by an increase in abundance of the resistant strain of *B. uniformis* JEB00174. Future research will be required to dissect the contributions of shifts in community function versus the altered behavior of the mutated host strain. In either case, we demonstrate that phages are capable of driving phenotypic and metabolic shifts in the microbiome.

A variety of synthetic communities modeling gut microbiota have been developed, including sFMT, hCom, SIHUMIx, and ASF (21, 23–25). With complexities ranging from 8 to 119 strains, each models a different facet of the intestinal microbiota. sFMT was originally designed to model the healthy microbiota in the absence of *Clostridioides difficile* colonization, while others, such as hCom, seek to capture a greater diversity of the most prevalent human gut microbes. As understanding of the role of phages in health and disease increases, it will be imperative to study their function in disease-relevant communities. A limitation of the approach deployed in this manuscript is that phages isolated from wastewater are likely to have been geographically and temporally isolated from their bacterial hosts. Given the strain specificity of phages and the constant arms race of predator and prey, future efforts should focus on paired isolation of phage and host from a single sample, which may provide a more evolutionarily relevant snapshot into their interactions. Furthermore, just as bacterial mono-colonization of the intestinal tract is improbable in a human, phages are generally found as diverse communities, which may introduce complex interactions and pressures on the microbial community, which must be studied in more depth.

Recent research into phage-host dynamics controlled by phase variation in the microbiome has focused on crAss-like phage and other species of *Bacteroides* (11, 12, 15). We describe phage-host dynamics for a novel Caudoviricetes phage in *B. uniformis*: a highly prevalent and abundant *Bacteroides* species in the human gut for which a similar understanding has been lacking. Phase variation represents an intriguing strategy for mediating predator-prey dynamics, providing rapid and reversible adaptation. It may be hypothesized that this use of preprogrammed heterogeneity ensures long-term association with phage and may, in some cases, have no apparent detriment to the host (12). Furthermore, phages may act as agents of horizontal gene transfer, providing an advantage to the host over the long term.

HKP09-induced modulation of sFMT metabolism exhibits several features of note, including amino acids and their metabolites. We observed an increase in 5-AVA, which is a product of Stickland fermentation of proline. We and others have previously reported proline fermentation as a key mediator of resistance against *C. difficile* infection (21, 26). We previously identified that one organism, *P. russellii* (*anaerobius*) JEB00254 is the dominant source of 5-AVA in this community (21), which was fitting with the observations that HKP09 infection altered *P. russellii* abundance in both gnotobiotic animal experiments. Other amino acids we identified play important roles in maintaining barrier function, immune homeostasis, and energy balance (27–30). HKP09 infection also altered the abundance of *C. scindens*, an organism best known for its ability to form secondary bile acid metabolites, which are of great importance in infectious and non-infectious diseases, including cancer, liver disease, obesity, and metabolic syndrome (31). Additional efforts must determine a causal role for these phage-induced metabolic shifts impacting host health, particularly in disease-relevant models. Given the experimental design of the *in vivo* experiments, it is not possible to disentangle the direct effects of HKP09 predation on *B. uniformis* versus potential indirect effects, including stimulating host immune responses, which is an area of active study (32). The hypothesis that phages could be used to reprogram the metabolism of communities, rather than simply knock out strains from a community as is typically the goal of phage therapy, has great translational and therapeutic potential but will require additional research to understand the biological consequences for host health.

Our research dissects the global impact a single phage has on a model gut microbial community. A limitation of this study was the use of a single phage, which is not representative of the typical highly diverse and little-understood human virome. Future studies will be needed to understand the additive effects of multiple phages and the mechanistic links between phage infection, microbe-microbe interaction networks, microbial metabolism, and host health. As the human virome/phageome field develops, experimental models and tools such as the ones we have reported will be essential to establish mechanisms and causation for associations between viral community composition and health.

## MATERIALS AND METHODS

### Isolation of bacteriophage

Bacteriophage HKP09 was isolated from sewage water acquired from the University Area Joint Authority Center Region Wastewater Treatment against *B. uniformis* DSM 6597. The sewage water was filtered using a 0.2 µm pore size membrane filter. The filtrate was combined with double-concentrated BHI CHV (74 g/L brain heart infusion [BHI], 0.10% wt/vol cysteine, 10 µg/mL hemin, 2 µg/mL menadione) and inoculated with a 1% inoculum of the target bacterial strain to enrich for bacteriophage that target *B. uniformis* DSM 6597. The phage enrichment was incubated at 37°C for 24 hours, then centrifuged (15 minutes, 6,000 × *g*, 4°C), and finally filtered using a 0.2 µm filter.

200 µL of filtered enrichment culture was mixed with 200 µL of *B. uniformis* DSM 6597 overnight culture. The mix was plated with a double-layer agar method using BHI top agar (37 g/L BHI, 7.5 g/L Agar). Single plaques were isolated and resuspended in 1 mL phage buffer (100 mM NaCl, 50 mM Tris-HCl [pH 7.4], 8 mM MgSO_4_). Phage suspension was purified by streaking on a BHI CHV plate and then letting it incubate at room temperature for 20 minutes. BHI top agar with 200 µL of overnight bacterial culture was overlaid and incubated at 37°C for 24 hours.

A plate lysate method was utilized to obtain a high-concentration phage lysate. To 100 µL of overnight liquid culture, 100 µL of 1E4-1E5 PFU/mL phage stock was mixed and plated using a double-layer agar overlay method. The plate was incubated at 37°C for 24 hours. The webbed plate was flooded with a phage buffer (100 mM NaCl, 50 mM Tris-HCl [pH 7.4], 8 mM MgSO_4_) and the surface was gently broken. The supernatant was centrifuged (8,000 × *g*, 4°C for 15 minutes) and filter-sterilized with a 0.2 µm size pore membrane filter.

### Bacteriophage genomic DNA extraction

The protocol for bacteriophage genomic DNA was adapted from the Center for Phage Technology protocol for extracting DNA from phage lysates using the Promega Wizard DNA Clean-Up System (cpt.tamu.edu/phage-links/phage-protocols/). For DNA extraction, 10 mL of bacteriophage lysate at a concentration (PFU/mL) of 1E10 PFU/mL was filter sterilized and then treated with 10 µL of 10 mg/mL DNase I and 10 µL of 10 mg/mL RNase A (final concentration for both is 10 µg/mL) for 30 minutes at 37°C. The phage was precipitated overnight with 5 mL of 20% PEG/2.5M NaCl solution at 4°C. The precipitated bacteriophage was centrifuged at 10,000 × *g* for 20 minutes at 4°C. The pellet was resuspended in 500 µL of 5 mM MgSO_4_. The resuspended pellet was treated with 2.5 µL of 20 µg/mL proteinase K and 10 µL of 0.5 M EDTA and was incubated at 50°C for 30 minutes.

The extraction and purification were done with the Promega Wizard kit. The phage suspension was thoroughly mixed with 1 mL of the Promega Wizard kit purification resin. The resin was previously incubated at 37°C and resuspended before usage. The suspension was filtered through the Wizard column using a 3 mL syringe. The column was subject to two rounds of washing with 80% isopropanol. The column was dried by centrifugation for 2 minutes at 13,000 *× g* at room temperature. The DNA was eluted from the column using 100 µL of preheated water and centrifugation for 1 minute at 13,000 × *g* at room temperature. The DNA was stored at −20°C. The phage genome was determined to be dsDNA on the basis of comparative quantification of dsDNA, ssDNA, and RNA using fluorometric assays (Qubit), and successful sequencing library preparations by tagmentation (Illumina DNA Prep) and Oxford Nanopore library preparation chemistry for dsDNA. The extracted DNA was sequenced using Oxford Nanopore Technology, performed by Plasmidsaurus.

### Phage genome annotation and classification

HKP09 genes were annotated via comparison against the PHROGs, CARD, and VFDB databases using Pharokka, which utilizes Phanotate for bacteriophage gene prediction (33). In addition, we queried taxonomy and homologous phages through comparative genomic analysis with vConTACT2 (16) against other sequenced phages in the Millard Lab Phage Genome May 2024 database (17). The database was filtered to include bacteriophages whose host was in the *Bacteroides* genus, given that HKP09 was experimentally confirmed to target *B. uniformis*. The network was created using vContact2 default filtering and clustering thresholds, wherein line weights representing –log(*P* value) connected to HKP09 were 63.2 [34.6–152.4], median [range]. Average nucleotide identity (ANI) of HKP09 against high-quality vOTU sequences of the UHGV Catalog was calculated using the anicalc.py script of CheckV (34). vOTUs with an alignment fraction >85% and an ANI > 90% were carried forward as representatives of HKP09, given the fuzzy boundaries of OTU generation. Viral mapping data and sample metadata were obtained from portal.nersc.gov/UHGV

### Co-culture growth assay of *B. uniformis* DSM 6597 and HKP09

Growth dynamics of *B. uniformis* DSM 6597 (JEB00023) were observed at a 10-fold dilution of bacteriophage. Liquid BHI CHV (37 g/L BHI, 15 g/L Agar, 0.05% wt/vol cysteine, 5 µg/mL hemin, and 1 µg/mL menadione) media was inoculated with 1% overnight culture and 1% of the phage dilution. A 96-well plate was run with triplicates incubated at 37°C with orbital mixing every 30 seconds and absorbance reading at 600 nm every 15 minutes. For the purposes of visualization, data were downsampled to show 30-minute intervals. All growth of strains was performed in an anaerobic chamber (Coy Anaerobic Systems, 5% H_2_, 20% CO_2_, 75% N_2_). After the co-culture growth assay, wells were subcultured onto BHI CHV plates and passaged two times. Liquid BHI CHV media was inoculated with 1% overnight cultures of the suspected mutant subcultures and then challenged with 1% of the phage dilutions as described in the co-culture assay. A 96-well plate was run with triplicate using the same plate reader settings.

### Spontaneous mutant DNA sequencing and analysis

Bacterial DNA was extracted from an overnight culture using an adjusted version of the ZymoBIOMICS Quick-DNA HMW MagBead Kit (D6060). Overnight culture was spun down at 5,000 × *g* for 1 minute, the pellet was washed in 1 mL of phosphate-buffered saline and spun at 5,000 × *g* for 1 minute. This pellet was resuspended in 100 µL of TE buffer and 25 µL of lysozyme (100 mg/mL) and incubated at 55°C for 30 minutes. After incubation, 20 µL of 10% SDS and 10 µL of proteinase K were added, followed by incubation at 55°C for 10 minutes. The sample was centrifuged at 5,000 × *g* for 1 minute to pellet residual debris. The supernatant was transferred to a new microcentrifuge tube. The DNA was then purified using the manufacturer’s instructions.

Short-read libraries were prepared using Illumina DNA preparation reagents and sequenced on an Illumina iSeq 100 with paired-end 150 nt reads. The same extracted DNA was sequenced on an Oxford Nanopore on an R10.4.1 flow cell following the v14 library prep method. Short and long reads were then aligned to the reference sequence for JEB00023 and analyzed for mutations and structural variants using VariantDetective (35). For phase inversions upstream of the promoter region for *upx*, Breseq (36) was used to find the rates of inversions with polymorphism mode enabled so that isolates were interpreted as mixed populations.

### Deletion of IRs

Products for the DNA sequences in between and flanking the two IRs in wild-type JEB00023 were amplified with Q5 DNA Polymerase (NEB) using the following primers: GATTAGCATTATGAGTGGATCCGTATTGCTGGATATTTATTTCGATTATTTGCTGGC, ACTTCCAAGTCAAAGTCATTTCCATTTATATATC, GGAAATGACTTTGACTTGGAAGTTCAAATTTCAGGAAATGAAATAGGGAATGAG, CAAAGATAAGACTGTTTTTTATATCCTCAAAATGT, GAGGATATAAAAAACAGTCTTATCTTTGGCTTTTTGTTTGTAATCCGTTCGTTTATAGTC, and GAAGATAGGCAATTAGTCGACTCTACAACACCAAGCGCCATATAG. The products were assembled using NEBuilder HiFi (NEB) with pLGB13 (37): a suicide vector designed for two-step allelic exchange using selective and non-selective markers, pre-digested with BamHI-HF and SalI-HF (NEB). The plasmid was verified by sequencing and conjugated into wild-type JEB00023 using *E. coli* strain S17-1. Erythromycin-resistant integrants were identified by PCR and plated on solid media containing 100 ng/mL anhydrotetracycline. Anhydrotetracycline-resistant strains were verified by sequential patching for loss of erythromycin resistance and by PCR. Two resulting strains, GT5316 and GT5317, were isolated and their sequences confirmed using Oxford nanopore long-read whole-genome sequencing, ensuring that there were no off-target mutations in paralogs of the same system. The results of sequencing also demonstrated other structural variations that occurred in the generated mutants relative to the wild type. Namely, in both constructed mutants, a group of phage-related proteins moved within the genome, though PHASTEST (38) did not label it as a phage. GT5316 and GT5317 observed large inversions and deletions for TonB and SusD proteins, as well as endonucleases. Of note, however, is that all of these structural variations occurred without the selective pressure of HKP09 infection. Analysis of SNPs or indels is not available for these strains as only long-read sequencing was performed.

### RT-qPCR of *upcY*

RNA was extracted using either the ThermoFisher Invitrogen PureLink RNA mini kit or QIAGEN RNeasy Mini Kit corresponding to Fig. 3 and 4, respectively. RNA was reverse transcribed using BioRad iScript Reverse Transcription Supermix for RT-qPCR or ThermoFisher H Minus cDNA Synthesis Kit with dsDNase for Fig. 3 and 4, respectively. The resulting cDNA was quantified using the following forward and reverse primers: GAGAAGGAGAACCTCGGCTG and TGACGGGAACCAAAGTACGG. Amplification was performed using a final primer concentration of 200 nM each and iTaqTM Universal SYBR Green Supermix. The cycling parameters were set to 95°C for 3 minutes and 40 cycles of 5 seconds at 95°C and 30 seconds at 60°C, with a lid temperature of 105°C. qPCR was performed on a BioRad CFX384 Opus. Abundance was quantified relative to the expression of *gapdh*, which was quantified using the same protocol and the following target-specific primers: CGATGTTCGTTTGCGGTGTT and GGAGCCAAGCAGTTGGTAGT.

### Phage spot titers

200 µL of host bacterial overnight culture was mixed with 3 mL of BHI top agar (37 g/L BHI, 7.5 g/L agar), and the mix was plated with a double-layer agar method using BHI top agar. Samples containing phage to be quantified were filtered through a 0.2 µm pore size membrane filter, and a 10-fold serial dilution was created. 2.5 µL of serial dilution per spot was dispensed onto the plate and incubated until a translucent bacterial lawn formed.

### Microbial culture and sFMT preparation

Bacterial strains were streaked onto BHI CHV (37 g/L BHI, 15 g/L agar, 0.05% wt/vol cysteine, 5 µg/mL hemin, and 1 µg/mL menadione). Strains were cultured in liquid variants of the same media without agar at 37°C anaerobically. For each strain, cells were pelleted by centrifugation and washed with fresh BHI CHV containing 15% glycerol prior to pooling at the equivalent optical density. The pooled communities were stored at −80°C freezer prior to *in vitro* assays and gnotobiotic mouse administration.

### *In vitro* sFMT assay

The assay was conducted in 30 mL of BHI CHV to which a 1% inoculum of the sFMT was added. Phage was added to the flask with a final concentration of 1E6 PFU/mL. At the time points, 1 mL aliquot from the flasks was centrifuged (2 minutes, 12,000 × *g*, RT). The bacterial pellet was separated from the supernatant. Both were stored in −80°C until extraction for sequencing or metabolomics.

### Gnotobiotic studies

Female C57BL/6J mice aged 9–12 weeks were housed with *ad libitum* water and food (Lab Diet 5021) under a 12-hour light/dark cycle. The experiment was replicated twice. In the first iteration of the experiment, mice were co-housed 4/cage by group; in the second iteration of the experiment, animals were singly housed. All animals were maintained inside Allentown Sentry Positive Pressure isolators at the PSU Gnotobiotics facility. Experiments were approved by an institutional review board (PSU IACUC #PROTO202101826). 1M sodium bicarbonate was orally administered 5 minutes prior to the sFMT. sFMTs were prepared by mixing 1 mL of prepared sFMT with 1 mL of bacteriophage or phage buffer prior to the administration of the oral gavage. Fecal samples were collected prior to the gavage at time 0, and then subsequently at 6 hours, 1 day, 2 days, and 7 days after the oral gavage.

### Amplicon sequencing

Microbial DNA and phage DNA were isolated using the ZymoBIOMICS 96 MagBead DNA kit (D4308). 200 µL of *in vitro* liquid samples or approximately 50 mg of frozen fecal samples was weighed and transferred into empty lysing tubes containing mixed-size zirconia beads (0.01–0.1 mm). 750 µL of ZymoBIOMICS lysis solution was added, then the slurry was disrupted for a total of 5 minutes on FastPrep96, and then centrifuged at 10,000 × *g* for 5 minutes. The DNA was then extracted and cleaned following the manufacturer’s instructions.

The V4 region of the 16S rRNA gene was amplified using the 515F (TCGTCGGCAGCGTCAGATGTGTATAAGAGACAGGTGYCAGCMGCCGCGGTAA) and 806R (GTCTCGTGGGCTCGGAGATGTGTATAAGAGACAGGGACTACNVGGGTWTCTAAT) primers. Full details of library preparation can be found at github.com/BisanzLab/OHMC_Colaboratory. Primary PCRs were conducted in 10 µL reactions with KAPA HiFi hot start enzyme and SYBR green to monitor reaction progress. Samples were subsequently diluted 100× before indexing with unique dual 10nt indexes based on the Illumina Tagmentation Sets A–D. Resulting indexed libraries were quantified with PicoGreen and pooled at equimolar concentrations. The library was size-selected by gel purification followed by Ampure bead cleanup using a 0.6× ratio. The DNA was sequenced on an Illumina NextSeq 2000 with XLEAP P1 600-cycle reagents for both *in vitro* and *in vivo* samples. The resulting data were processed using v2.1 of the following script: github.com/BisanzLab/OHMC_Colaboratory/blob/main/analysis_scripts/AmpliconSeq_q2.Rmd. Briefly, reads were processed using QIIME2 version 2023.5, primer sequences were removed, allowing for an error rate of 0.15, discarding any read without a valid primer sequence on both ends. Next, reads were denoised and overlapped using Dada2. Taxonomy was assigned using the Dada2 taxonomic classifier against the SILVA version 138.1 database. Amplicon sequence variants (ASVs) were matched to sFMT members through pairwise alignment with their respective 16S rDNA sequence, allowing for one mismatch. Strains that could not be separated based on the ambiguity in mapping of ASVs are marked as such in their respective figures. 16S gene amplicon sequencing of the *in vitro* experiment resulted in 72,479 ± 36,124 (mean ± sd) reads/sample after QC and denoising. 16S gene amplicon sequencing of the gnotobiotic experiment resulted in 140,213 ± 60,891 (mean ± sd) reads/sample after QC and denoising.

To quantify inversion at the UpcY locus *in vivo*, custom primers were developed pairing target-specific primers with partial overhangs for i5 and i7 adapters: TCGTCGGCAGCGTCAGATGTGTATAAGAGACAGGCTGGGTTTTATCTTTATGATGGGT and GTCTCGTGGGCTCGGAGATGTGTATAAGAGACAGGCAGTTCGTCATTACCTTCTGG. Samples were indexed and sequenced as above. Data were trimmed, denoised, and filtered using Dada2. After QC and denoising, 23,426 ± 18,496 (mean ± sd) upx reads/sample were obtained. The resulting ASVs were mapped to sequences of JEB00023 or JEB00174’s UpcY locus (in both inversion states), and the best-scoring alignments were used to classify the ASVs, with a required minimum percent identity of 97%. Frequencies of the ASVs in sequencing data were subsequently used to calculate relative rates of inversion.

### Metagenomic sequencing

gDNA was extracted from fecal pellets using a DNeasy 96 PowerSoil Pro QIAcube HT Kit with liquid handling performed by an Integra mini-96 1250 µL multichannel and a QIAcube HTP extraction platform. Full details of sample extraction and library preparation can be found at github.com/BisanzLab/OHMC_Colaboratory. Samples were normalized to 6 ng/µL on the basis of fluorimetric quantification (Quant-iT), and 6 µL was used as the input to a 1/5th scale Illumina DNA Prep protocol using unique dual indexes based on the Illumina Tagmentation Sets A–D. The resulting library was balanced using an Illumina iSeq100 2 × 150 run before full sequencing on a paired-end 150 NovaSeq X 25B lane (Novogene USA). Samples were sequenced with 43,196,663 ± 5,747,337 (mean ± sd) reads. Strain abundances were determined using StrainR2 with default parameters as previously described (22). All input strains were detected in the input community administered to mice. KEGG ortholog abundances were determined by extracting KOs from each reference genome using KOFamScan. Community KO abundances were determined as the sum of each genome’s abundance (wpFUKM) multiplied by that genome’s KO copy number. Differential KO abundances were determined by Welch’s t-test on log_2_-transformed abundances with a significance threshold of *P* < 0.05. Pathway enrichment was performed using ClusterProfiler (39) with a Benjamini-Hochberg false discovery rate threshold of 0.1.

### qPCR of *B. uniformis* strains and HKP09

The absolute abundance of HKP09, *B. uniformis* DSM 6597, and *B. uniformis* JEB00174 (4_1_36) was quantified using qPCR. Primers were generated *in silico* and then confirmed *in vitro* using gDNA from pure cultures. HKP09 forward primer: TTGCGAAGCAGAAAGACCCC, HKP09 reverse primer: GAACACCTGCCCATTATCGC. JEB00023 forward primer: ACTCTACGACTTGACTGCCC, JEB00023 reverse primer: GCTCTGTGCTATGGAGAGGG. JEB00174 forward primer: TGGAGTTCGGCGTAGCTTTT, JEB00174 reverse primer: TCTCGGCATTCCAACCAGAC. For sample amplification, each primer had a final concentration of 200 nM, and iTaqTM Universal SYBR Green Supermix was used. The BioRad CFX384 Opus thermocycler was set to an initial denaturation of 95°C for 3 minutes with 40 cycles of 5 seconds at 95°C and 30 seconds at 60°C. Absolute abundance was calculated using a standard curve of pure gDNA of target samples and then normalized to sample mass or volume.

### ^1^H NMR-based metabolomics

Extraction of approximately 50 mg of feces was performed using 1.2 mL of 0.1 M phosphate buffer (K_2_HPO_4_/NaH_2_PO_4_ = ½, pH = 7.4, 50% D_2_O). An internal standard of 0.005% wt per volume of sodium 2-(trimethylsilyl) propionate-2,2,3,3-d4 (TSP) was used. Samples were subject to vortexing and two freeze-thaw cycles and then centrifuged for 10 minutes at 18,213 × *g* (≥18,000 × *g*) and 4°C. 550 µL of the clear supernatant in a 5 mm NMR tube was used in subsequent NMR analysis. ^1^H spectra of sample extracts were recorded using a Bruker Avance NEO 600 MHz NMR spectrometer (Bruker Biospin). Spectra were analyzed using Chenomx NMR Suite (v.12.0). Metabolites were identified and then quantified by comparing to the known concentration of the internal standard (TSP). The final concentrations were normalized to sample weight (µmol/g). Z-scores were calculated on a per-metabolite basis within experiments. A sample from one mouse in the first gnotobiotic experiment was removed due to an abnormally high detection of lactate and a low detection of all other metabolites. Data were visualized using principal components analysis (prcomp, R). Differential abundance analysis was performed by Welch’s t-test on Z-score normalized data.

## Data Availability

Sequencing data and relevant metadata have been deposited to the NCBI Sequence Read Archive under project accession PRJNA1298916.

## SUPPLEMENTAL MATERIAL

Supplemental material mbio.02341-25-s0001.pdf Fig. S1 to S4; Tables S1 and S2.

ASM does not own the copyrights to Supplemental Material that may be linked to, or accessed through, an article. The authors have granted ASM a non-exclusive, world-wide license to publish the Supplemental Material files. Please contact the corresponding author directly for reuse.
