# Supplementary material for: A synthetic gut microbiota provides an understanding of the maintenance and functional impact of phage
Source: mBio. 2025 Nov 12;16(12):e02341-25. doi: 10.1128/mbio.02341-25 (PMC12691588; doi:10.1128/mbio.02341-25)
Supplement: Supplemental material — Fig. S1 to S4; Tables S1 and S2. [file mbio.02341-25-s0001.pdf]

Supplemental data for: A synthetic gut microbiota provides understanding of the maintenance and functional impact of phage

Heejung Koo[a], Kerim Heber[a], Shuchang Tian[a], Shane T. Connolly[a], Fuhua Hao[b], Jingcheng Zhao[a], Bethany Swencki-Underwood[a], Andrew D. Patterson[a,b,c], Guy E. Townsend[c,d], Jordan E. Bisanz[a,c]#

[a] Department of Biochemistry and Molecular Biology, Pennsylvania State University, University Park, PA, USA
[b] Department of Veterinary and Biomedical Sciences, Center for Molecular Toxicology and Carcinogenesis, The Pennsylvania State University, University Park, PA, USA
[c] One Health Microbiome Center, Huck Life Sciences Institute, University Park, PA, USA
[d] Department of Molecular and Precision Medicine, Penn State College of Medicine, Hershey, PA, USA
# Address correspondence to Jordan Bisanz, jordan.bisanz@psu.edu
H. Koo and K. Heber contributed equally to this work. Author order was determined based on contribution to project conception.

SUPPLEMENTAL FIGURES

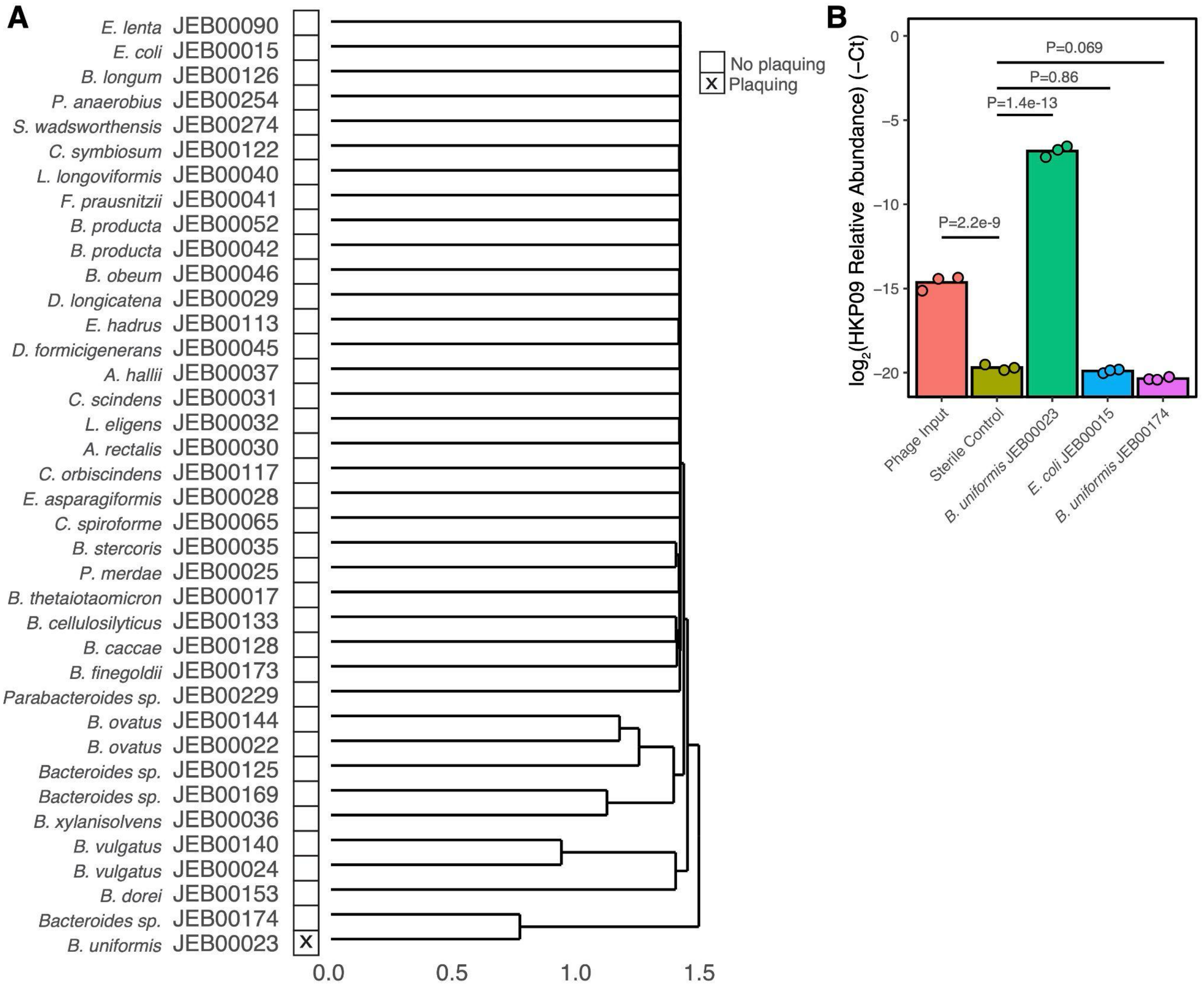

Figure S1. HKP09 only productively infects *B. uniformis* JEB00023. (A) Results of a plaquing assay on each of the sFMT strains. Strains are organized by whole genome phylogeny determined using PhyloPhlan. **(B)** qPCR quantification of viral replication on select strains including *B. uniformis* JEB00174. Strains were challenged with 1e6 PFU HKP09 and grown for 24h at 37˚C in anaerobic conditions. In the absence of a host strain, a significant decrease in HKP09 abundance was noted which was not significantly different from *B. uniformis JEB00174*, or negative control *E. coli.* As anticipated, the viral abundance of HKP09 propagated on host *B. uniformis* JEB00023 increased 7,643-fold compared to the sterile control. Statistical analysis by ANOVA with Tukey's HSD.

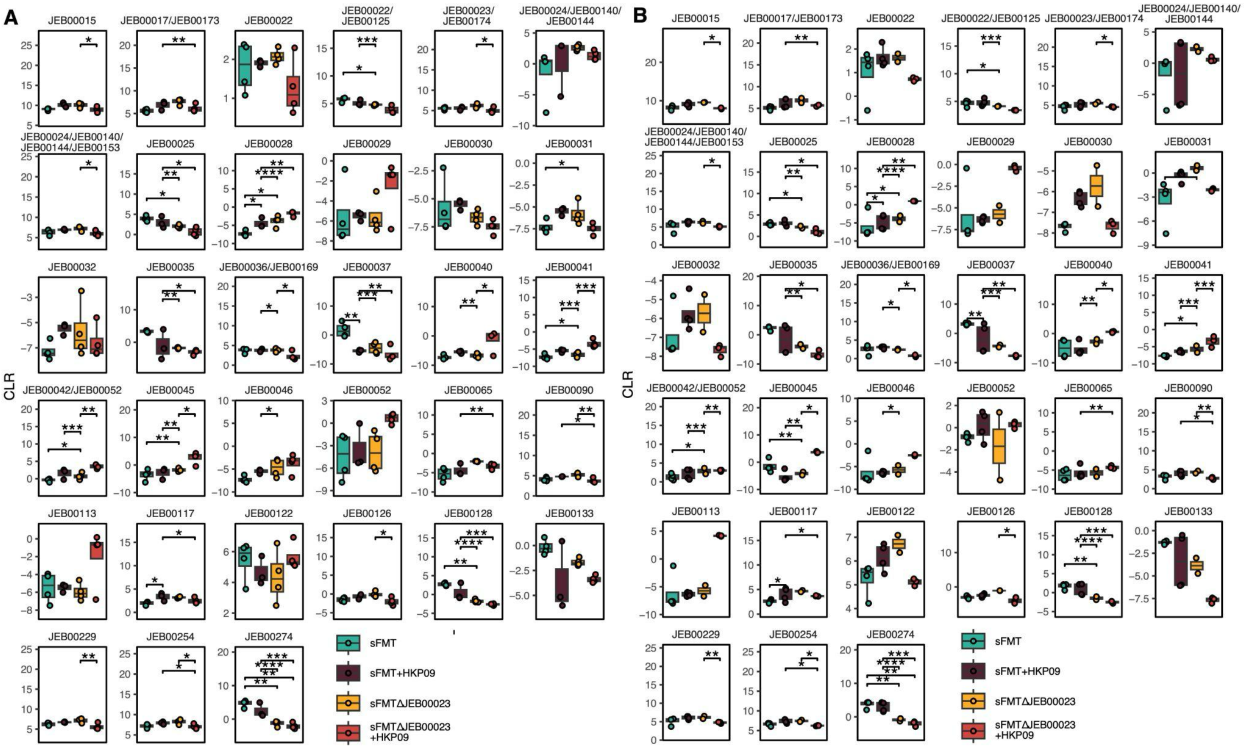

Figure S2. Differential abundance of all sFMT strains *in vitro* over time. 16S rRNA amplicon sequencing of *in vitro* experiments at **(A)** 1 day and **(B)** 2 days. Statistical analysis by ANOVA with Tukey HSD. * denotes a significance of less than 0.05, ** denotes a significance of less than 0.01, and *** denotes a significance of less than 0.001. CLR, centered $\log_2$ ratio.

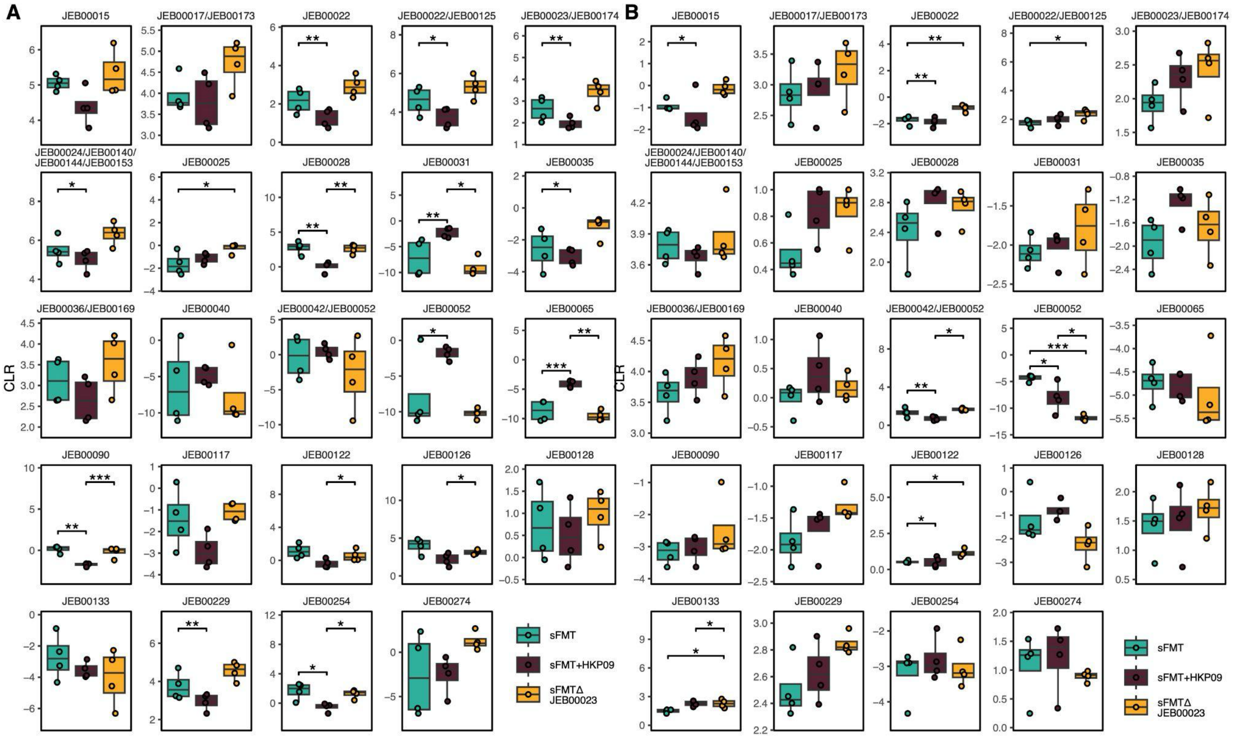

Figure S3. Differential abundance of sFMT strains *in vivo* over time (16S rRNA sequencing). Sequencing of gnotobiotic mouse fecal samples collected at **(A)** 2 days and **(B)** 7 days indicate that community composition was most varied between groups at 2 days, whereas differing abundances were less commonly observed at 7 days. Statistical analysis by ANOVA with Tukey HSD. * denotes a significance of less than 0.05, ** denotes a significance of less than 0.01, and *** denotes a significance of less than 0.001.

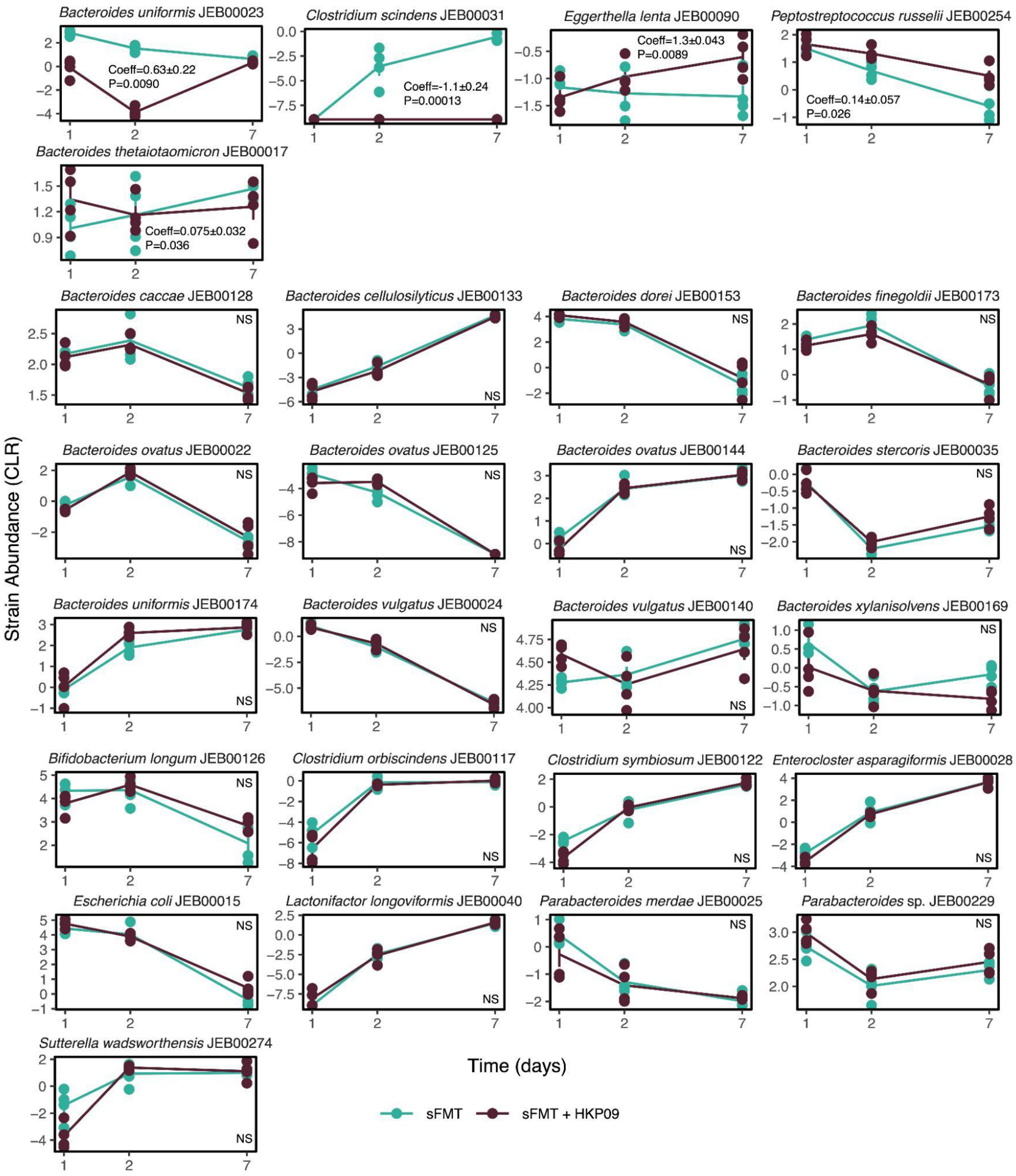

Figure S4. Differential abundance of sFMT strains *in vivo* over time (metagenomic sequencing). 5 strains exhibited significant effects of HKP treatment (top rows). N=4 mice/group, statistical analysis by linear mixed effects model extracting interaction of time and group.

SUPPLEMENTAL TABLES

Table S1. sFMT strains and genome accessions

LabID	Species	Strain ID	Genome Accession
JEB00015	*Escherichia coli*	DSM 18039	GCA_000005845.2
JEB00017	*Bacteroides thetaiotaomicron*	DSM 2079	GCA_014131755.1
JEB00022	*Bacteroides ovatus*	DSM 1896	GCA_001314995.1
JEB00023	*Bacteroides uniformis*	DSM 6597	JBEUMQ000000000
JEB00024	*Bacteroides vulgatus*	DSM 1447	GCA_000012825.1
JEB00025	*Parabacteroides merdae*	DSM 19495	GCA_900445495.1
JEB00028	*Enterocloster asparagiformis*	DSM 15981	GCA_000158075.1
JEB00029	*Dorea longicatena*	DSM 13814	GCA_000154065.1
JEB00030	*Agathobacter rectalis*	DSM 17629	GCA_000209935.1
JEB00031	*Clostridium scindens*	DSM 5676	GCA_004295125.1
JEB00032	*Lachnospira eligens*	DSM 3376	GCA_000146185.1
JEB00035	*Bacteroides stercoris*	DSM 19555	GCA_900106605.1
JEB00036	*Bacteroides xylanisolvens*	DSM 18836	GCA_000210075.1
JEB00037	*Anaerobutyricum hallii*	DSM 3353	GCA_000173975.1
JEB00040	*Lactonifactor longoviformis*	DSM 17459	GCA_002915525.1
JEB00041	*Faecalibacterium prausnitzii*	DSM 17677	GCA_010509575.1
JEB00042	*Blautia producta*	DSM 3507	GCA_002915535.1
JEB00045	*Dorea formicigenerans*	DSM 3992	GCA_000169235.1
JEB00046	*Blautia obeum*	DSM 25238	GCA_000153905.1
JEB00052	*Blautia producta*	DSM 2950	GCA_010669205.1
JEB00065	*Clostridium spiroforme*	DSM 1552	GCA_000154805.1
JEB00090	*Eggerthella lenta*	DSM 2243	GCA_003339945.1
JEB00113	*Eubacterium hadrus*	DSM 3319	GCA_000332875.2
JEB00117	*Clostridium orbiscindens*	1_3_50AFAAA	GCA_000760655.1
JEB00122	*Clostridium symbiosum*	WAL-14673	GCA_000189615.1
JEB00125	*Bacteroides ovatus*	D2	GCA_000159075.2
JEB00126	*Bifidobacterium longum*	35B	GCA_000261225.1
JEB00128	*Bacteroides caccae*	CL03T12C61	GCA_000273725.1
JEB00133	*Bacteroides cellulosilyticus*	CL02T12C19	GCA_000273015.1
JEB00140	*Bacteroides vulgatus*	CL09T03C04	GCA_000273295.1
JEB00144	*Bacteroides ovatus*	3_8_47FAA	GCA_000218325.1
JEB00153	*Bacteroides dorei*	CL03T12C01	GCA_000273075.1
JEB00169	*Bacteroides xylanisolvens*	2_1_22	GCA_000162155.1
JEB00173	*Bacteroides finegoldii*	CL09T03C10	GCA_000304195.1
JEB00174	*Bacteroides uniformis*	4_1_36	GCA_000185585.1
JEB00229	*Parabacteroides sp.*	D13	GCA_000162275.1
JEB00254	*Peptostreptococcus russellii [anaerobius]*	CC14N	SAMN42012254
JEB00274	*Sutterella wadsworthensis*	HGA0223	GCA_000411515.1

Table S2. Differentially abundant KEGG Orthologous Groups

KO	log2(FC)	P value	Strains with Gene Family	Annotation
K15870	-3.64	0.025	JEB00031	baiCD; 3-oxocholoyl-CoA 4-desaturase [EC:1.3.1.115]
K15871	-3.64	0.025	JEB00031	baiF; bile acid CoA-transferase [EC:2.8.3.25]
K15873	-3.64	0.025	JEB00031	baiH; 7beta-hydroxy-3-oxochol-24-oyl-CoA 4-desaturase [EC:1.3.1.116]
K09002	-3.64	0.025	JEB00031	csm3; CRISPR-associated protein Csm3
K18783	-3.64	0.025	JEB00031	E2.4.1.279; nigerose phosphorylase [EC:2.4.1.279]
K24707	-3.64	0.025	JEB00031, JEB00042	E2.7.1.16; ribulokinase [EC:2.7.1.16]
K00862	-3.64	0.025	JEB00031	eryA; erythritol kinase (D-erythritol 1-phosphate-forming) [EC:2.7.1.215]
K17204	-3.64	0.025	JEB00031, JEB00037	eryE; erythritol transport system ATP-binding protein
K17203	-3.64	0.025	JEB00031, JEB00037	eryF; erythritol transport system permease protein
K17202	-3.64	0.025	JEB00031, JEB00037	eryG; erythritol transport system substrate-binding protein
K25156	-3.64	0.025	JEB00031, JEB00042, JEB00052	evrA; viologen exporter family transport system ATP-binding protein
K25155	-3.64	0.025	JEB00031, JEB00042, JEB00052	evrB; viologen exporter family transport system permease protein
K25154	-3.64	0.025	JEB00031, JEB00042, JEB00052	evrC; viologen exporter family transport system permease protein
K25067	-3.64	0.025	JEB00029, JEB00031, JEB00042, JEB00045, JEB00052	fcsSBP; fucose transport system substrate-binding protein
K09681	-3.64	0.025	JEB00030, JEB00031, JEB00113	gltC; LysR family transcriptional regulator, transcription activator of glutamate synthase operon
K00261	-3.64	0.025	JEB00031	GLUD1_2, gdhA; glutamate dehydrogenase (NAD(P)+) [EC:1.4.1.3]
K07069	-3.64	0.025	JEB00031	K07069; uncharacterized protein
K07219	-3.64	0.025	JEB00031, JEB00037	K07219; putative molybdopterin biosynthesis protein
K09116	-3.64	0.025	JEB00031, JEB00037, JEB00042, JEB00046, JEB00052, JEB00113	K09116; damage-control phosphatase, subfamily I [EC:3.1.3.-]
K16907	-3.64	0.025	JEB00031, JEB00042	K16907; fluoroquinolone transport system ATP-binding protein [EC:7.6.2.-]
K25068	-3.64	0.025	JEB00031, JEB00042, JEB00052	K25068; fucose transport system permease protein
K25069	-3.64	0.025	JEB00031, JEB00042, JEB00052	K25069; fucose transport system permease protein
K00917	-3.64	0.025	JEB00031, JEB00046, JEB00113	lacC; tagatose 6-phosphate kinase [EC:2.7.1.144]
K10188	-3.64	0.025	JEB00031, JEB00042, JEB00046, JEB00052	lacE, araN; lactose/L-arabinose transport system substrate-binding protein
K10189	-3.64	0.025	JEB00031, JEB00042, JEB00046, JEB00052	lacF, araP; lactose/L-arabinose transport system permease protein
K10190	-3.64	0.025	JEB00031, JEB00042, JEB00046, JEB00052	lacG, araQ; lactose/L-arabinose transport system permease protein
K26115	-3.64	0.025	JEB00031, JEB00113	lctB; lactate dehydrogenase (NAD+,ferredoxin) subunit LctB
K16179	-3.64	0.025	JEB00029, JEB00031, JEB00045	mtbC; dimethylamine corrinoid protein
K00899	-3.64	0.025	JEB00031	mtnK; 5-methylthioribose kinase [EC:2.7.1.100]
K09697	-3.64	0.025	JEB00031, JEB00065	natA; sodium transport system ATP-binding protein [EC:7.2.2.4]
K00918	-3.64	0.025	JEB00031	pfkC; ADP-dependent phosphofructokinase/glucokinase [EC:2.7.1.146 2.7.1.147]
K03298	-3.64	0.025	JEB00031	TC.DME; drug/metabolite transporter, DME family
K23356	-3.64	0.025	JEB00029, JEB00031, JEB00037, JEB00045, JEB00113	trmB; HTH-type transcriptional regulator, sugar sensing transcriptional regulator
K05772	-3.64	0.025	JEB00031	tupA, vupA; tungstate transport system substrate-binding protein
K05773	-3.64	0.025	JEB00031	tupB, vupB; tungstate transport system permease protein
K08364	-2.95	2.00E-04	JEB00023, JEB00133	merP; periplasmic mercuric ion binding protein
K24258	-1.43	6.49E-05	JEB00023, JEB00024, JEB00025, JEB00035, JEB00065	wbjD; UDP-N-acetyl-L-fucosamine synthase [EC:5.1.3.28]
K18431	-1.19	3.75E-04	JEB00023, JEB00024, JEB00169	legF, ptmB; CMP-N,N'-diacetyllegionaminic acid synthase [EC:2.7.7.82]
K09459	-0.56	5.12E-05	JEB00017, JEB00023, JEB00035, JEB00052, JEB00122	E4.1.1.82; phosphonopyruvate decarboxylase [EC:4.1.1.82]
K23999	-0.56	5.12E-05	JEB00017, JEB00023, JEB00035, JEB00122	fom1; phosphoenolpyruvate phosphomutase / 2-hydroxyethylphosphonate cytidylyltransferase [EC:5.4.2.9 2.7.7.104]
K19068	-0.29	1.34E-03	JEB00017, JEB00023, JEB00024, JEB00025, JEB00029, JEB00035, JEB00045, JEB00065, JEB00128, JEB00173, JEB00174	wbjC; UDP-2-acetamido-2,6-beta-L-arabino-hexul-4-ose reductase [EC:1.1.1.367]

K09988	-0.23	3.25E-04	JEB00023, JEB00025, JEB00029, JEB00031, JEB00040, JEB00042, JEB00046, JEB00052, JEB00128, JEB00133, JEB00229	lyxA; D-lyxose ketol-isomerase [EC:5.3.1.15]
K19170	-0.22	0.043	JEB00028, JEB00036, JEB00173	dndC; DNA sulfur modification protein DndC
K01191	-0.19	0.026	JEB00017, JEB00023, JEB00042, JEB00052, JEB00133, JEB00144, JEB00174	MAN2C1; alpha-mannosidase [EC:3.2.1.24]
K15894	-0.19	0.002	JEB00017, JEB00023, JEB00024, JEB00025, JEB00035, JEB00045, JEB00052, JEB00065, JEB00090, JEB00122, JEB00128, JEB00133, JEB00173, JEB00174, JEB00229	pseB, fnlA, wbjB; UDP-N-acetylglucosamine 4,6-dehydratase [EC:4.2.1.115]
K15915	0.35	0.028	JEB00025, JEB00133, JEB00174, JEB00229	pglC; undecaprenyl phosphate N,N'-diacetylbacillosamine 1-phosphate transferase [EC:2.7.8.36]
K09931	0.36	0.048	JEB00031, JEB00045, JEB00254	K09931; uncharacterized protein
K01259	0.36	0.048	JEB00029, JEB00031, JEB00254	pip; proline iminopeptidase [EC:3.4.11.5]
K12999	0.36	0.048	JEB00030, JEB00031, JEB00045, JEB00046, JEB00065, JEB00113, JEB00254	rgpI; glucosyltransferase [EC:2.4.1.-]
K15023	0.38	0.045	JEB00040, JEB00042, JEB00052, JEB00254	acsE; 5-methyltetrahydrofolate corrinoid/iron sulfur protein methyltransferase [EC:2.1.1.258]
K03740	0.38	0.042	JEB00040, JEB00042, JEB00052, JEB00090, JEB00254	dltD; D-alanine transfer protein
K10793	0.38	0.036	JEB00029, JEB00031, JEB00040, JEB00254	prdA; D-proline reductase (dithiol) PrdA [EC:1.21.4.1]
K10794	0.38	0.036	JEB00029, JEB00031, JEB00040, JEB00254	prdB; D-proline reductase (dithiol) PrdB [EC:1.21.4.1]
K05593	0.41	0.034	JEB00029, JEB00041, JEB00052, JEB00133, JEB00174	aadK; aminoglycoside 6-adenylyltransferase [EC:2.7.7.-]
K14983	0.41	0.031	JEB00090, JEB00254	ciaR; two-component system, OmpR family, response regulator CiaR
K03739	0.41	0.031	JEB00042, JEB00052, JEB00090, JEB00254	dltB; membrane protein involved in D-alanine export
K07533	0.41	0.031	JEB00090, JEB00254	prsA; foldase protein PrsA [EC:5.2.1.8]
K02825	0.41	0.031	JEB00090, JEB00254	pyrR; pyrimidine operon attenuation protein / uracil phosphoribosyltransferase [EC:2.4.2.9]
K19244	0.45	0.017	JEB00254	ala; alanine dehydrogenase [EC:1.4.1.1]
K02116	0.45	0.017	JEB00030, JEB00032, JEB00113, JEB00254	atpI; ATP synthase protein I
K19090	0.45	0.017	JEB00037, JEB00254	cas5t; CRISPR-associated protein Cas5t
K19091	0.45	0.017	JEB00037, JEB00254	cas6; CRISPR-associated endoribonuclease Cas6 [EC:3.1.-.-]
K03399	0.45	0.017	JEB00254	cbiE; cobalt-precorrin-7 (C5)-methyltransferase [EC:2.1.1.289]
K23774	0.45	0.017	JEB00254	ccpN; DeoR family transcriptional regulator, catabolite repression regulator
K05311	0.45	0.017	JEB00254	cggR; central glycolytic genes regulator
K05884	0.45	0.017	JEB00065, JEB00254	comC; L-2-hydroxycarboxylate dehydrogenase (NAD+) [EC:1.1.1.337]
K19137	0.45	0.017	JEB00029, JEB00254	csn2; CRISPR-associated protein Csn2
K19075	0.45	0.017	JEB00037, JEB00254	cst2, cas7; CRISPR-associated protein Cst2
K07067	0.45	0.017	JEB00254	disA; diadenylate cyclase [EC:2.7.7.85]
K01819	0.45	0.017	JEB00254	E5.3.1.26, lacA, lacB; galactose-6-phosphate isomerase [EC:5.3.1.26]
K13607	0.45	0.017	JEB00254	fldA; cinnamoyl-CoA:phenyllactate CoA-transferase [EC:2.8.3.17]
K03436	0.45	0.017	JEB00052, JEB00113, JEB00254	fruR2, fruR; DeoR family transcriptional regulator, fructose operon transcriptional repressor
K13693	0.45	0.017	JEB00254	gpgS; glucosyl-3-phosphoglycerate synthase [EC:2.4.1.266]
K00260	0.45	0.017	JEB00254	gudB, rocG; glutamate dehydrogenase [EC:1.4.1.2]
K20025	0.45	0.017	JEB00254	hadB; (R)-2-hydroxyisocaproyl-CoA dehydratase alpha subunit [EC:4.2.1.157]
K05967	0.45	0.017	JEB00113, JEB00254	K05967; uncharacterized protein
K06993	0.45	0.017	JEB00029, JEB00030, JEB00032, JEB00037, JEB00046, JEB00065, JEB00113, JEB00254	K06993; ribonuclease H-related protein

K15976	0.45	0.017	JEB00254	K15976; putative NAD(P)H nitroreductase [EC:1.-.-.-]
K00520	0.45	0.017	JEB00254	merA; mercuric reductase [EC:1.16.1.1]
K17883	0.45	0.017	JEB00254	mtr; mycothione reductase [EC:1.8.1.15]
K13995	0.45	0.017	JEB00254	nicF; maleamate amidohydrolase [EC:3.5.1.107]
K02594	0.45	0.017	JEB00254	nifV; homocitrate synthase NifV [EC:2.3.3.14]
K16651	0.45	0.017	JEB00254	pduX; L-threonine kinase [EC:2.7.1.177]
K22305	0.45	0.017	JEB00254	psp; phosphoserine phosphatase [EC:3.1.3.3]
K09685	0.45	0.017	JEB00254	purR; HTH-type transcriptional regulator, purine operon repressor
K05985	0.45	0.017	JEB00041, JEB00065, JEB00254	rnmV; ribonuclease M5 [EC:3.1.26.8]
K14154	0.45	0.017	JEB00254	THI6; thiamine-phosphate diphosphorylase / hydroxyethylthiazole kinase [EC:2.5.1.3 2.7.1.50]
K00984	0.50	0.010	JEB00174	aadA; streptomycin 3''-adenylyltransferase [EC:2.7.7.47]
K15896	0.50	0.010	JEB00174	pseH; UDP-4-amino-4,6-dideoxy-N-acetyl-beta-L-altrosamine N-acetyltransferase [EC:2.3.1.202]
